# Unexpected Redox Chemistry of P∩N- and As∩N-Rhenium(I) Tricarbonyl Complexes in the Presence of CO_2_ Acting as an Acid

**DOI:** 10.1021/acs.inorgchem.3c02925

**Published:** 2023-10-06

**Authors:** Martin Ertl, Uwe Monkowius, Kerstin T. Oppelt

**Affiliations:** † Linz School of Education—Chemistry, Johannes Kepler University Linz, Altenberger Strasse 69, 4040 Linz, Austria; ‡ Department of Chemistry, University of Zürich, Winterthurerstrasse 190, 8057 Zürich, Switzerland

## Abstract

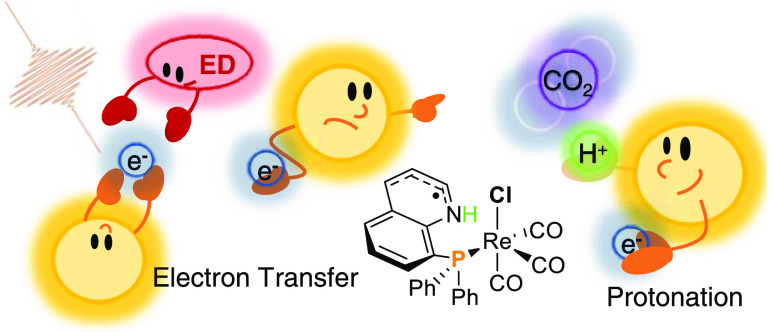

This study reports on Re tricarbonyl complexes bearing 8-(diphenylphosphanyl)quinoline, P∩N, and 8-(diphenylarsanyl)quinoline, As∩N, as bidendate ligands. We studied the reactivity of these complexes in comparison with *fac*-Re(N∩N)(CO)_3_Cl (with N∩N = 2,2′-bipyridine or 4,4′-dimethyl-2,2′-bipyridine). We used a combination of electrochemical and spectroelectrochemical methods with time-resolved spectroscopy over 10 orders of magnitude (100 ps–1 s) to investigate the peculiar reactivity of one-electron-reduced Re(CO)_3_(P∩N)Cl and Re(CO)_3_(As∩N)Cl complexes also in the presence of protons.

## Abstract 2

This study reports on the reactivity of Re tricarbonyl complexes bearing 8-(diphenylphosphanyl)quinoline, P∩N, and 8-(diphenylarsanyl)quinoline, As∩N, complexes in comparison with *fac*-Re(N∩N)(CO)_3_Cl (with N∩N = 2,2′-bipyridine or 4,4′-dimethyl-2,2′-bipyridine). We combined electrochemical and spectroelectrochemical methods with time-resolved spectroscopy covering time scales of 10 orders of magnitude to enable the time resolution of both intra- and intermolecular reactions of the singly reduced complex.

## Introduction

Rhenium tricarbonyl complexes are widely investigated compounds in the fields of photochemical and photophysical research. Their best known application in the area of artificial photosynthesis and light to chemical energy conversion is the electro- and photochemical reduction of CO_2_ to CO and/or formate. Since the beginning of the studies on Lehn’s catalyst, i.e., *fac*-Re(bpy)(CO)_3_Cl (with bpy = 2,2′-bipyridine)^1,2^ modifications of substituents and ligands have led to deeper understanding of the catalytic activity of Re(bpy)(CO)_3_Cl and related systems. Alongside systematic molecular modifications, spectroscopic methods combined with electrochemistry were developed to investigate reaction pathways of these metal carbonyl complexes.^3−5^

The ability of the carbonyl vibrational modes to report on the oxidation state of the Re metal center have been utilized in many mechanistic studies.^4,6^ The combination of a moderate absorbance in the visible region and high absorbance of their carbonyl vibrational modes around ν_CO_ 1850–2100 cm^–1^ make them ideal for steady state, *in situ*, and transient infrared spectroscopic methods.^7^

**Scheme 1:**
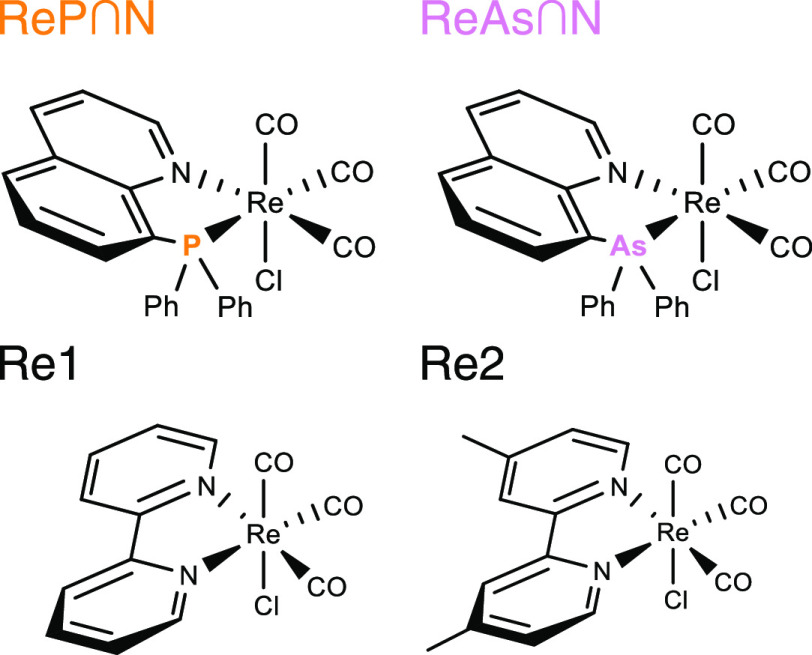
Molecular Structures of Rhenium Tricarbonyl Complexes Addressed in This Work: **ReP**∩**N**, **ReAs**∩**N** (Re(CO)_3_(P∩N)Cl, Re(CO)_3_(As∩N)Cl), this work. **Re1** (Re(bpy)(CO)_3_Cl)^1,2^ and **Re2** (Re(dmbpy)(CO)_3_Cl)^3^

The rhenium(I) tricarbonyl complexes under investigation are depicted in Scheme 1. **ReP**∩**N** and **ReAs**∩**N** contain the ligands 8-(diphenylphosphanyl)quinoline and 8-(diphenylarsanyl)quinoline, respectively. **Re1** and **Re2** represent complexes bearing 2,2′-bipyridine (bpy) and 4,4′-dimethyl-2,2′-bipyridine (dmbpy), respectively. The latter two complexes have been thoroughly investigated and are thus selected as reference compounds.^3−5^ Both complex types ReE∩N (E = pnictogens P or As) and ReN∩N feature a chelating five-membered ring motif containing the rhenium center with the main difference that for the ReE∩N complexes, the coordinating pnictogen E is not part of the π-system of the aromatic backbone like it is the case for the diimine N∩N-type ligands.

For photochemical applications in general, a long electronically excited-state lifetime of the photoactivated molecule is beneficial because this increases the chances for electron transfer to the excited molecule from a suitable reducing agent. **Re1**, **Re2**, and related compounds have shown excited-state lifetimes that can be somewhat limiting for application in many photochemical CO_2_ and proton reduction systems.^6,8−11^ Our new compounds show a much longer excited-state lifetime (see below), which motivated us to carry out more detailed photochemical and photophysical studies.

Previous studies show two main ways where ligand variation can influence optical absorption and emission properties of tricarbonyl complexes derived from Re(bpy)(CO)_3_Cl:^12,13^ the axial position and the chelating ligand. Phosphine^14−16^ or phosphine oxide,^17^ relevant to this study, often targeted the axial position.^5^ Phosphine then replaces the halide ligand^5,18^ or even the CO ligand trans to the halide,^18^ forming a cis-dicarbonyl complex in the latter case. Modifications in the chelating ligands, like in our case, have been investigated as well. The energy of the ππ*-transition of the diimine can be shifted downward, relative to the MLCT transition, through higher conjugation^19^ or by changing this typical ππ*-transition to an intraligand charge transfer.^20,21^ Compared to Re tricarbonyl complexes with diimine ligands, there is much less literature regarding photophysics of complexes with P∩N ligands. However, recent literature also shows application of Re dicarbonyl complexes with bis(diphenylphosphino)amine ligands (additional to bipyridine ligands) for CO_2_ reduction.^22^ Additionally, the photophysics of complexes with P–C–N^23^ (2-(diphenylphosphanyl)pyridyl ligand) or P–N–C–N (*N*-(diphenylphosphanyl)pyridin-2-amine) has been investigated.^24^ While the latter ligand has a similar binding motif to the Re center as in **ReP**∩**N**, the less extended π-system of the described pyridyl-based ligands, compared to quinoline ligands (as in **ReP**∩**N** and **ReAs**∩**N**), may entail much different photophysics between the two different ligand systems.

Following the investigation of basic photophysics of **ReP**∩**N** and **ReAs**∩**N**, we studied the electrochemistry and resolved the reaction kinetics of these complexes. Initial results in the presence of CO_2_ suggested unusual CO_2_ binding after 1-electron reduction. Instead, however, a quite different but equally unusual reactivity could be identified.

## Results

### Molecular Structures of **ReP**∩**N** and **ReAs**∩**N**

**Scheme 2:**
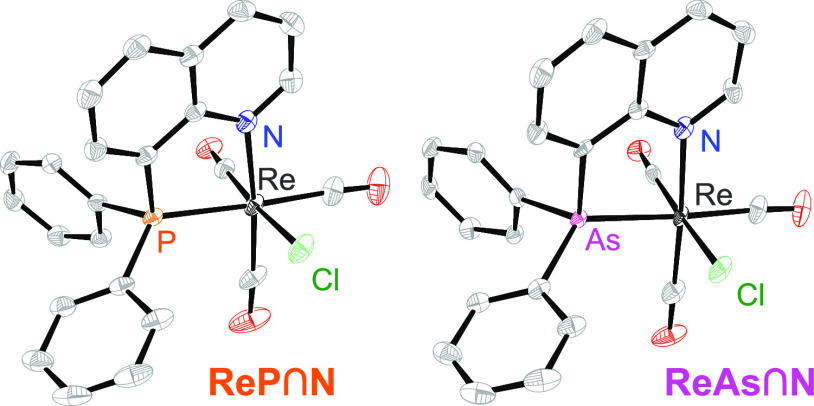
Molecular Structures of **ReP**∩**N** and **ReAs**∩**N**

**ReP**∩**N** and **ReAs**∩**N** complexes are isostructural and crystallize in monoclinic space group *P*2_1_/*n*. Thus, it is not surprising that the crystallographic parameters are very similar: the rhenium center exists in a pseudo-octahedral environment with three carbonyl ligands in a facial configuration. Scheme 2 shows the molecular structures of **ReP**∩**N** and **ReAs**∩**N**.

The P/As- and N-atoms of the bidentate E∩N ligand and two carbonyl ligands lie in the same plane, also including the quinoline moiety. Consequently, the chloride ligand is perpendicular to this plane. As expected, the Re–P bond (2.4396(7) Å) is significantly shorter than the Re–As bond (2.5319(7) Å). The Re–Cl bond lengths are very similar (P: 2.508(1) Å; As: 2.499(1) Å) and so are the Re–N bond lengths (P: 2.230(3) Å; As: 2.251(4) Å). Also the bite angles of the ligands E–Re–N are practically identical (P: 79.19(6)°; As: 79.11(9)°). The Re–C bond lengths lie between 1.916(3) and 1.957(4) Å with Re–C bonds slightly longer trans of the coordinating pnictogen E. These values resemble rhenium complexes bearing bpy-type ligands.^9^

### Photophysical Properties

**Figure 1:**
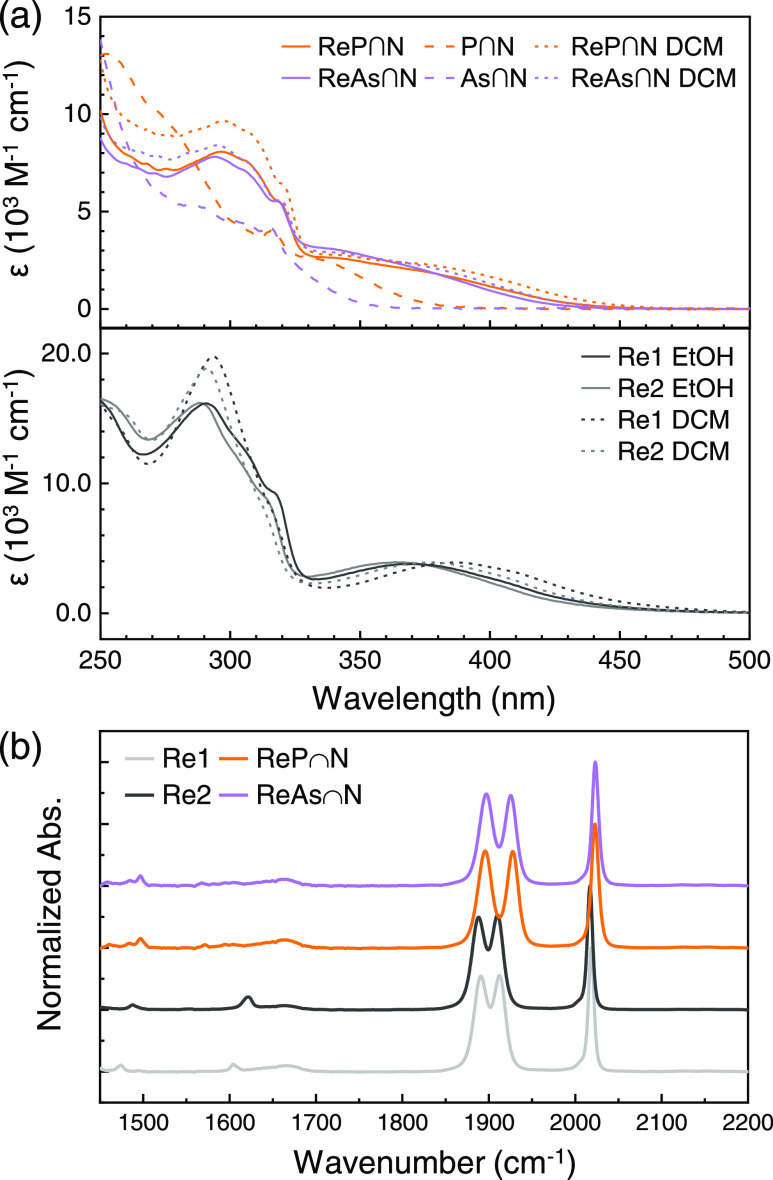
(a) UV–vis absorption spectra of all four investigated complexes in ethanol (EtOH) (solid) and dichloromethane (DCM) (dotted). The spectra of the free ligands (scattered) have been measured in ethanol. (b) FT-IR spectra of all four complexes in dimethyl sulfoxide (DMSO) normalized to the absorbance maximum of their respective a′(1) ν_CO_ modes.

Figure 1a shows the UV–visible absorption spectra of both **ReP**∩**N** and **ReAs**∩**N**, compared to the one of **Re1** and **Re2** in dimethylformamide. For **Re1** and **Re2**, the MLCT transition is around 340–440 nm.^20^ The corresponding spectral features of **ReP**∩**N** and **ReAs**∩**N** are blue-shifted compared to **Re1** and **Re2** and less resolved from the intraligand (IL) transitions at higher energy (see Figure 1a). **ReP**∩**N** and **ReAs**∩**N** show the typical three carbonyl vibrational modes in the FT-IR spectrum of *fac*-arranged CO ligands, which can be identified by relatively equal transition dipole moments of the three modes^25^ (see Figure 1b).

Phosphorescence lifetimes of the molecules are summarized in Table 1. The unstructured emission of the rhenium tricarbonyl complexes with 8-(diphenylphosphanyl)quinoline ligands have strong ^3^MLCT character, and thus, the excited-state lifetime is expected to be sensitive to the presence of oxygen as well as to solvent polarity. The P∩N and As∩N ligands seem to enable rather long excited-state lifetimes at room temperature compared to the diimine ligands of **Re1** and **Re2** (Table 1).^20^ This makes them ideal candidates for photochemical reduction studies. The collective frequency upshift of the tricarbonyl vibrational modes in the electronically excited state (see the subsection Time-Resolved Infrared Spectroscopy) also supports an MLCT charge transfer character of the lowest energy electronic excitation, as increased back bonding is a sign for a decrease of electron density at the Re center.

**Table 1:** Summary of Phosporescence Lifetime Data of **ReP**∩**N** and **ReAs**∩**N**

sample	λ_max_, τ at RT (solvent)^a^	λ_max_, τ at 77 K (solvent glass)^a^
**ReP**∩**N**	645 nm, 1.6 μs (CH_2_Cl_2_)	
	635 nm, 680 ns (EtOH)	560 nm, 100 μs (EtOH)
**ReAs**∩**N**	635 nm, 2.7 μs (CH_2_Cl_2_)	
	630 nm, 530 ns (EtOH)	555 nm, 75 μs (EtOH)
**Re1**	620 nm, 45 ns (CH_2_Cl_2_)	
	620 nm, 24 ns (EtOH)	530 nm, 2.8 μs (EtOH)
**Re2**	610 nm, 58 ns (CH_2_Cl_2_)	
	610 nm, 28 ns (EtOH)	520 nm, 3.0 μs (EtOH)

a Estimated errors are ±10%.

With the aid of quantum chemical calculations (Figure S1), the excited-state properties of the tricarbonyl complexes with halogen ligands can be characterized further. The lowest energy excitation of **Re1** and **Re2** is sometimes described as mixed metal to ligand/halide ligand to ligand charge transfer MLCT/LL’CT, i.e., the MLL’CT process.^26^ This is reasoned by local electron density on both the Re center and the axial ligand in the HOMO. For **ReP**∩**N** and **ReAs**∩**N**, this is similar (Figure S1). The HOMOs are located on the Re atom as well as the chloride ligands, however, with a slight contribution of electrons at the chelating ligand already in the HOMO of **ReP**∩**N** and **ReAs**∩**N**. So one can describe the lowest energy transition in **ReP**∩**N** and **ReAs**∩**N** as XMLCT (X stands for the halide ligand).

### Electrochemistry and Spectroelectrochemistry

**Figure 2:**
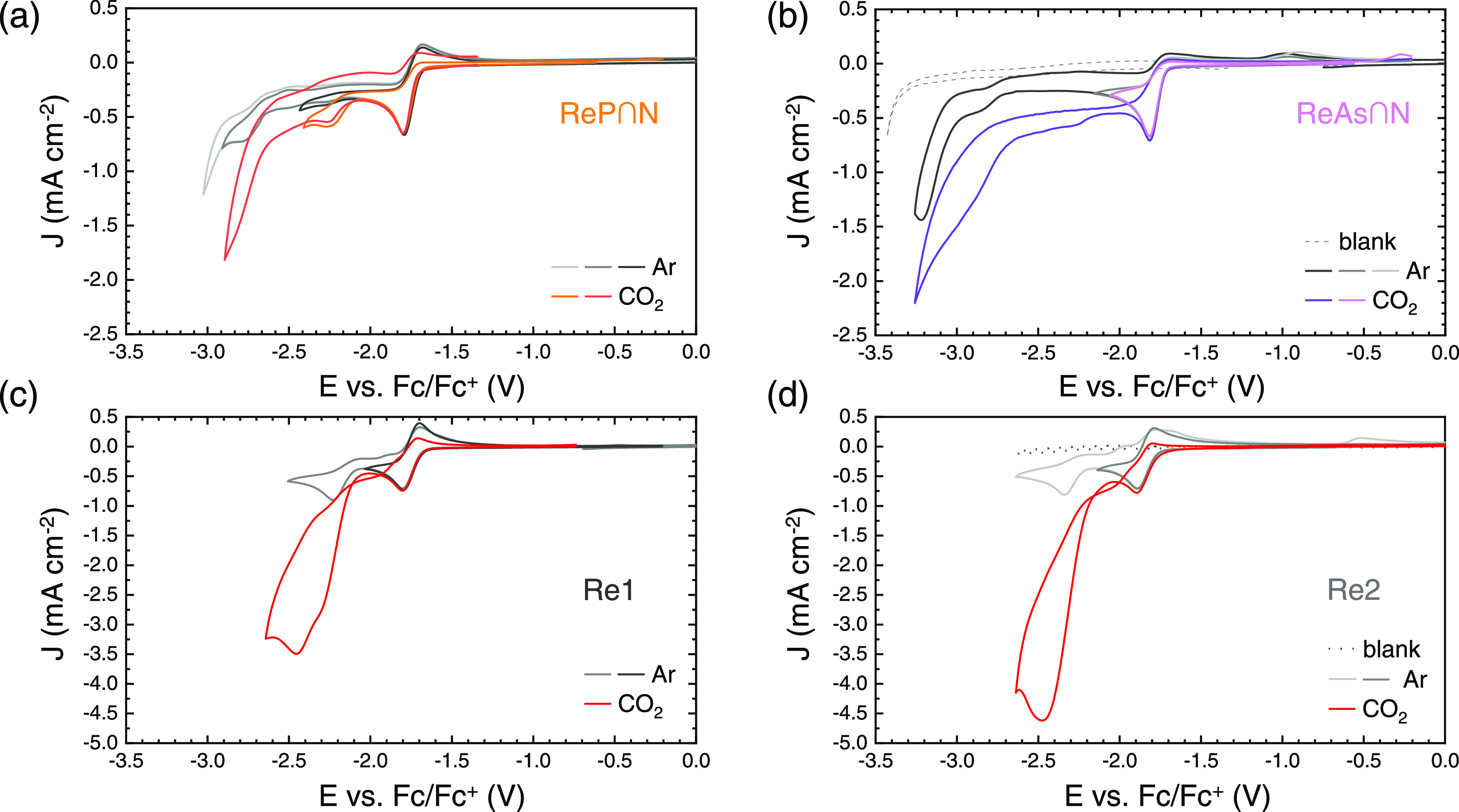
Cyclic voltammograms of the investigated complexes in DMF/0.1 M TBAPF_6_ with/without CO_2_ at 50 mV s^–1^ scan rate. (a) CV of **ReP**∩**N**. The measurements cover different potential ranges of the reduction of **ReP**∩**N** under an Ar (gray lines) or CO_2_ atmosphere (colored lines). (b) Analogous experiment but with **ReAs**∩**N**. A blank was measured for Ar (black, dashed) saturated electrolyte. (c, d) Similar measurements for **Re1** and **Re2**, respectively.

Cyclic voltammetry of **ReP**∩**N** and **ReAs**∩**N**, as well as **Re1** and **Re2**, was performed in dimethylformamide (DMF) with 0.1 M tetrabutylammonium hexafluorophosphate TBAPF_6_ as a conducting electrolyte (Figure 2). The first wave for **ReP**∩**N** appears around *E*_1/2_ ≈ −1.750 V vs Fc/Fc^+^, which is between the first reduction of **Re1** and **Re2**, so **Re1** is easier to reduce than **ReP**∩**N** and **Re2** with electron-donating methyl substituents on the dmbpy ligand is a little harder to reduce than **ReP**∩**N**. The first reduction of **ReAs**∩**N** appears to be roughly 20 mV more negative than for **ReP**∩**N**. This is in accordance with their calculated relative LUMO levels (Figure S1). Single-electron reduction/reoxidation can also be a method to determine the reversibility of the first electron transfer and is usually informative regarding the stability of the Re–Cl bond in Re-diimine complexes such as **Re1** and **Re2**. For [Re(P∩N)(CO)_3_Cl]^•–^ and [Re(As∩N)(CO)_3_Cl]^•–^, a similar analysis was performed (see first reductive waves in Figure 2). While the CV waves of the first reduction for **Re1** are narrow and the peak current ratio is truly unity, reduction of **ReP**∩**N** is not fully reversible under the applied conditions at 50 mV s^–1^ scan rate, where—similar to **Re2**—the anodic peak current is ca. 10–20% smaller than the cathodic one. This asymmetry is even more pronounced for **ReAs**∩**N**. Figure S2 compares the normalized first reductive waves of all four species.

Full reductive scans of the investigated complexes are shown in Figure 2 in an Ar (gray lines) and CO_2_ atmosphere (colored lines). Scans were reversed at different potentials to separately investigate the reductive waves. The separation of the first and second reduction potentials in **ReP**∩**N** and **ReAs**∩**N** is much larger than that in **Re1** and **Re2**. Instead of a catalytic wave at the second reduction potential, an additional peak around −2.7 V appears in the presence of CO_2_ for the new compounds and the first reduction peak becomes even more irreversible.

At equal concentrations (5.0 ± 0.2 mM) and scan conditions, all 4 complexes show a comparable peak current density of 0.75 mA cm^–1^ (Figure 2a–d). This indicates that the first reductive wave is due to a single-electron transfer for all four compounds. Additionally, **Re1** and **ReP**∩**N** have a constant ratio of the anodic peak current to the cathodic peak current of the Fc/Fc^+^ redox couple, i.e., the peak current ratio *i*_pc_(Re)/*i*_pa_(Fc) remains constant at different scan rates. This can be best seen upon normalization of the respective CV scans to the maximum of the anodic Fc/Fc^+^ peak (Figure S3).

**Figure 3:**
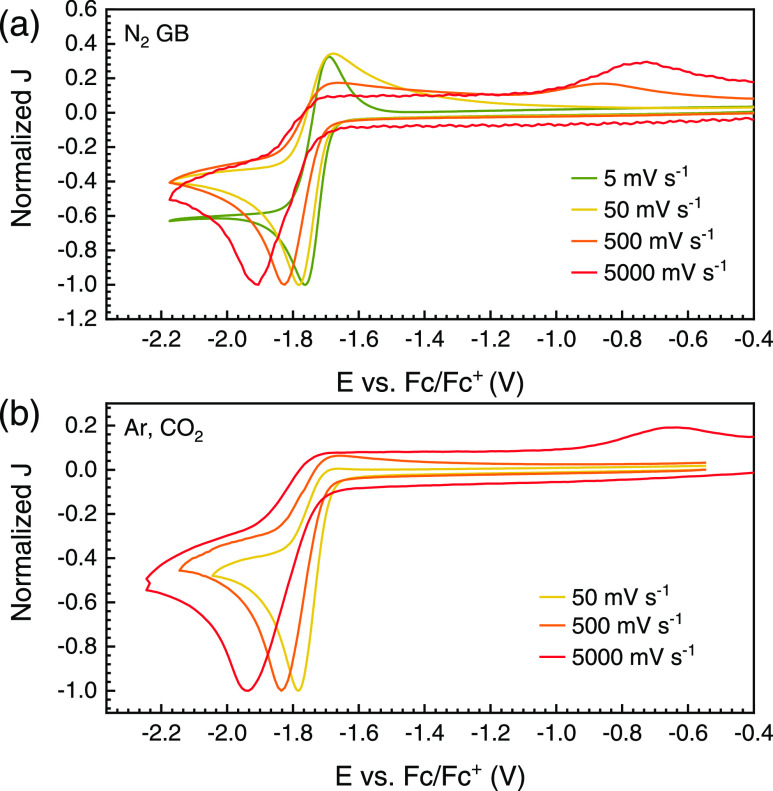
Normalized cyclic voltammograms of **ReP**∩**N** in DMF/0.1 M TBAPF_6_ (with or without CO_2_) at different scan rates. (a) First reduction of **ReP**∩**N** measured in a N_2_-filled glovebox (GB) without CO_2_. (b) First reduction of **ReP**∩**N** measured under blanket gas after bubbling with CO_2_ (4.5) with a maximum of 5 ppm water.

Figure 3a shows the (normalized) CVs for **ReP**∩**N** at different scan rates, which have a significant effect on the reversibility of the first reductive wave. The first reduction is almost completely reversible at very slow scan rates, while at fast scan rates, a new oxidative peak at −0.7 V is observed instead of the expected half-wave at −1.7 V. This may be explained with an EC_rev_ mechanism, i.e., a reversible chemical reaction after the first electron transfer, where one of the reduction products has a more positive reduction potential than the original complex.^27^ Excess of Cl^–^ (with or without cooling) had no influence (Figure S4) nor did a change of solvent (DMSO, DCM) (Figures S4 and S5). This points toward an intramolecular reversible chemical step after the first reduction rather than a possible loss (and readdition) of coordinated Cl^–^.

We found that the reductive wave remained irreversible even at the slower scan rate of 50 mV s^–1^ in the presence of CO_2_ (see Figure 3b), suggesting an intermolecular reaction which perturbs the intramolecular EC_rev_ equilibrium.

Both species formed in the reversible EC_rev_ reaction as well as the molecules formed in the presence of CO_2_ should be observable by spectroelectrochemistry (SEC).

**Figure 4:**
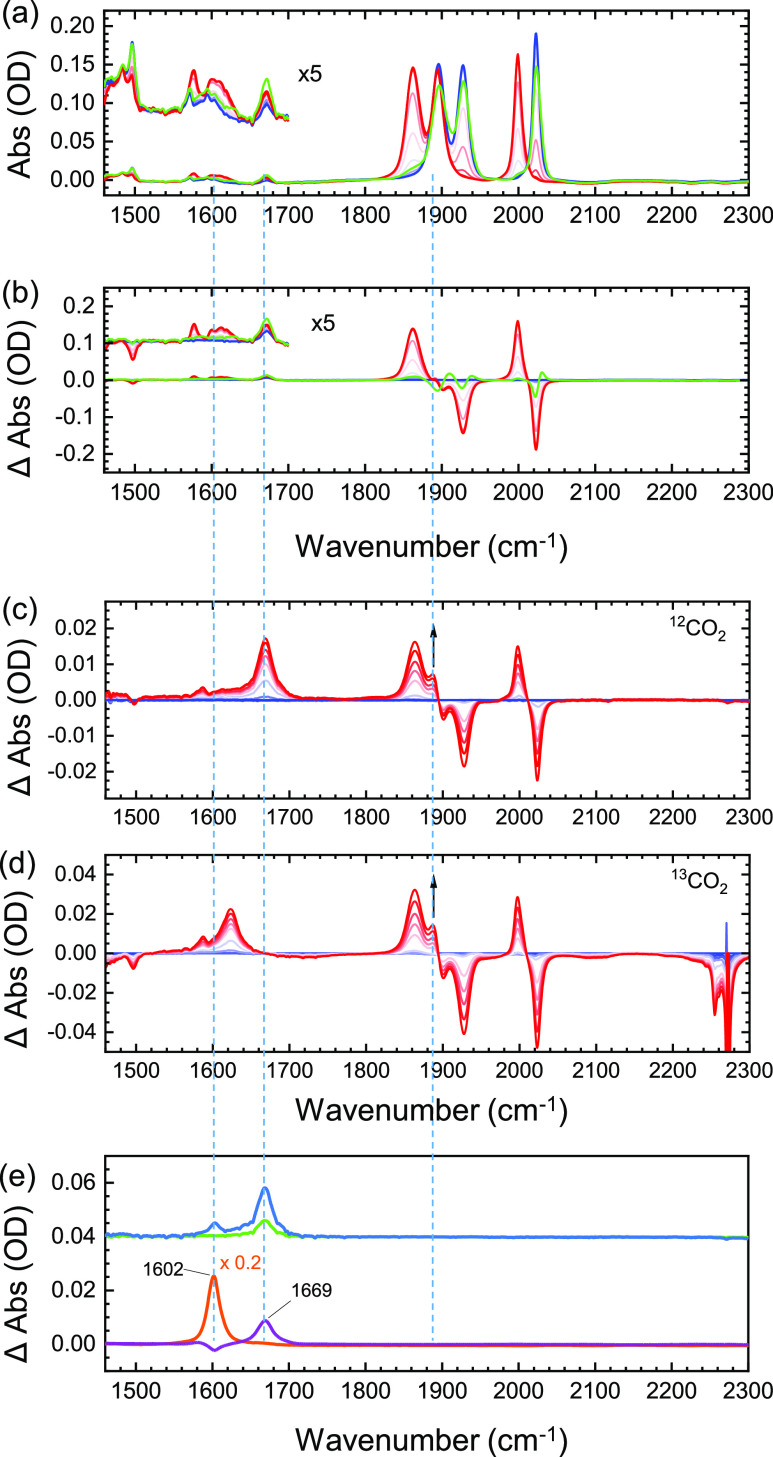
Spectroelectrochemistry of **ReP**∩**N** in DMSO (0.3 M TBAPF_6_): (a) FT-IR absorption spectra taken during a CV scan at 2 mV s^–1^ from 0 V (blue) to −1.8 V (red) vs Fc/Fc^+^ under Ar, showing close to 100% conversion. The green line is the last spectrum after returning to the initial potential. (b) Difference spectra of (a). (c) Difference spectra under ^12^CO_2_ during stepwise controlled potential electrolysis (CPE) from 0 V (0–60 s) to −1.6 V (60–240 s). (d) Same as panel (c) but with the isotope-labeled ^13^CO_2_. (e) Absorption of ^12^CO_2_ alone in dry DMSO after 120 s at −1.6 V (green) and after another 120 s at −1.90 V (blue) and reference spectra of saturated solutions of sodium formate (orange) and potassium hydrogen carbonate (violet).

At −1.7 V vs Fc/Fc^+^ under Ar in DMSO (see Figure 4a), the three carbonyl stretching modes near 2000 cm^–1^ shift to lower frequency (blue line vs red line), indicating decreased back bonding upon reduction of the P∩N ligand, similar to **Re1** and **Re2**.^3^ The initial spectrum (blue) is largely recovered upon reversing the potential scan (final spectrum: green line in Figure 4a), confirming the reversibility of the electrochemical reaction. When the measurement is repeated in the presence of CO_2_ (Figure 4c), additional positive bands appear at 1875 cm^–1^ and in the 1500–1700 cm^–1^ region. Surprisingly, the new peak in the CO-stretch region at 1875 cm^–1^ remains unchanged when isotope-labeled ^13^CO_2_ is used instead of ^12^CO_2_ (Figure 4d). We would expect this band to isotopically shift if it was due to bound CO_2_. It should thus be assigned to a carbonyl mode that responds to a change in the electron density at the Re center. Likewise, a small band at 1586 cm^–1^ shows no isotope shift. We assign it to a change in the ring modes of the ligands. A similar band is also present without CO_2_ at 1577 cm^–1^ (Figure 4a). On the other hand, the large peak forming at 1669 cm^–1^ in the presence of ^12^CO_2_ undergoes a strong isotope shift to 1623 cm^–1^. It can be partly assigned to the formation of hydrogen carbonate by comparison to the spectrum of a solution of KHCO_3_ in DMSO (red line in Figure 4e). HCO_3_^–^ was also observed to form at the electrodes in the absence of **ReP**∩**N**, albeit in a smaller amount (green curve in Figure 4d). This signal increases at a more negative potential (−1.9 V, blue line in Figure 4e). The smaller additional peak forming at this potential at 1603 cm^–1^ does not coincide with any of the peaks found in the presence of **ReP**∩**N** at −1.6 V, but it can be assigned to formate by comparison to the spectrum of a solution of HCOONa in DMSO (orange trace in Figure 4e).

The formation of HCO_3_^–^ and formate requires the presence of water, although measurements were performed under dry conditions. However, we observed a continuous increase in H_2_O absorption in the OH-stretch region upon CO_2_ bubbling (see the Supporting Information). Clearly, the purity of the gas (4.5) was not sufficient to prevent water accumulation. As a result, the perturbation of the EC_rev_ equilibrium and the spectral changes in the CO stretching and ligand ring modes may be due to the presence of additional protons.

**Figure 5:**
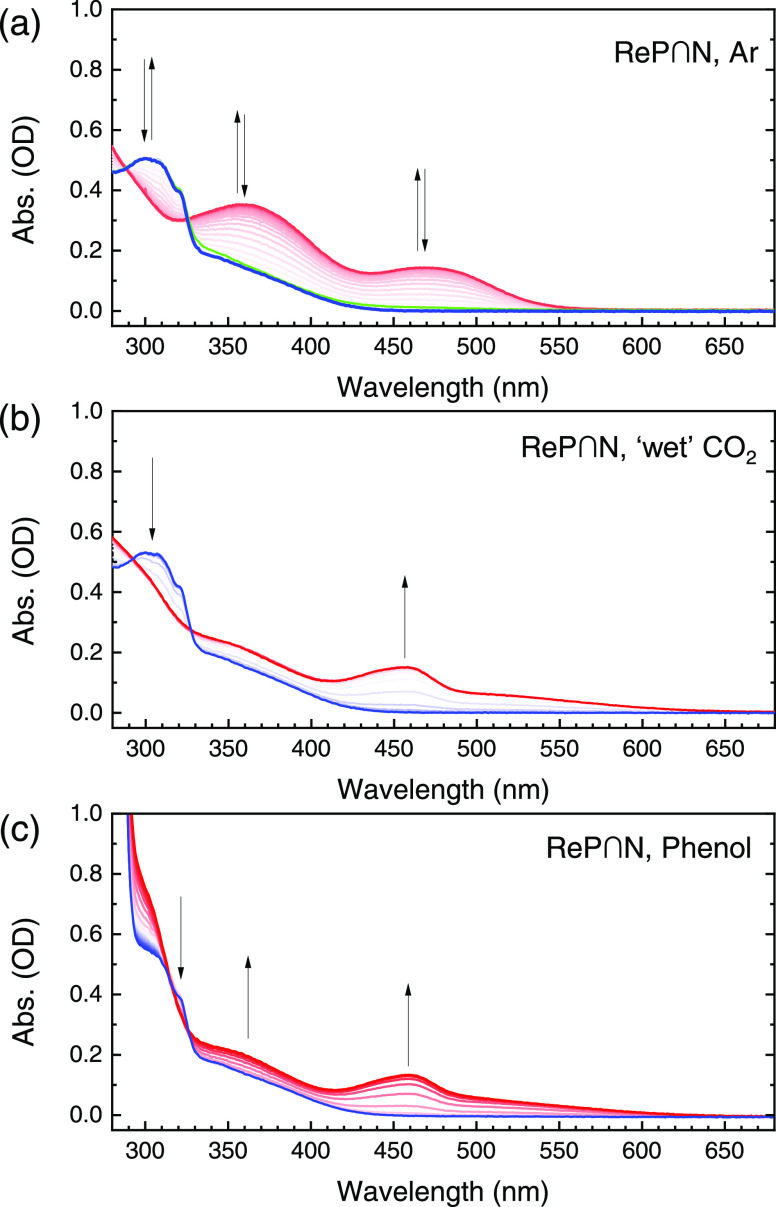
UV–vis spectroelectrochemistry of (a) **ReP**∩**N** under Ar in DMSO (0.1 M TBAPF_6_), while scanning a CV of the first reductive peak at 2 mV s^–1^. (b) Same as **ReP**∩**N** with “wet” ^12^CO_2_ in DMSO/0.1 M TBAPF_6_ but only the spectral data for the reductive sweep is shown. (c) Same as panel (b) but with phenol as a proton source.

**Figure 6:**
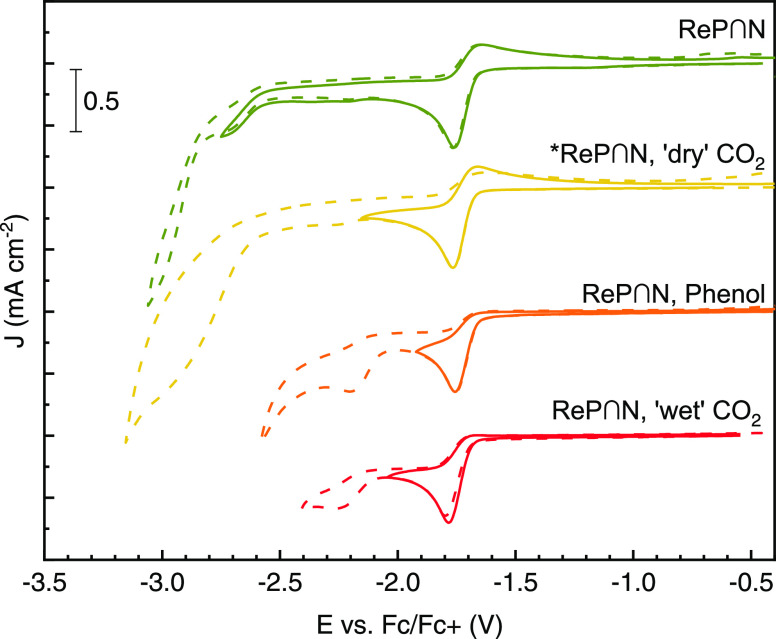
CV of 5 mM **ReP**∩**N** with 0.3 M phenol (and/or CO_2_) at 50 mV s^–1^. The data marked with * are from different data sets; thus, the current density was scaled by a factor of 1.2 as well as *E* values shifted by 20 mV to match the peak current/position of the first reduction peak.

We supplemented the measurements in the FT-IR regions with SEC in the visible region, where the effect of alternative proton sources could be more easily investigated (Figure 5). The UV–vis data confirm that the single-electron reduction is fully reversible in the absence of CO_2_ (compare blue vs green line in Figure 5a) and at least partially reversible in its presence. However, the spectral changes are very distinct. Furthermore, addition of 0.3 M phenol instead of CO_2_ bubbling produces strikingly similar absorption bands (Figure 5b vs c). The question was if this observation also translates to the cyclic voltammogram. Indeed, the CV scan in the presence of phenol and “wet” CO_2_, i.e., CO_2_ directly from the cylinder, in Figure 6, are almost identical.

A very similar CV response is observed in the presence of low concentrations of trifluoroacetic acid (p*K*_a_ = 0.52)^28^ (see Figure S7). In the presence of “wet” CO_2_, there is a more pronounced second reductive peak at −2.2 V vs Fc/Fc^+^ (Figure 2a), possibly due to a protonated and hence neutral compound **ReP**∩**N-H** that is easier to reduce than **ReP**∩**N**^•–^. Additional CO_2_ in the 0.3 M phenol solution does not change the CV at intermediate scan rates (50 mV s^–1^). Only at slow scan rates (5 mV s^–1^), the presence of both phenol and “wet” CO_2_ leads to a further increase of the peak current of the second reduction at −2.2 V. Carbonic acid (p*K*_a_ = 6.35)^28^ is a stronger acid than phenol (p*K*_a_ = 9.99),^28^ so the effect of CO_2_ on **ReP**∩**N**^•–^ is most likely just a hidden pH response. Most importantly, bubbling carefully dried CO_2_ had no effect on the CV of **ReP**∩**N**^•–^.

In summary, the singly reduced **ReP**∩**N** and **ReAs**∩**N** complexes are significantly more sensitive to protons in the electrolyte than singly reduced **Re1** and **Re2**, as a result of an unusual EC_rev_ mechanism, which we will now address in more detail (Table 2).

**Table 2:** Electrochemical and IR Spectral Data of **ReP**∩**N** and **ReAs**∩**N** (a) from FT-IR-SEC (b) from trIR

	*E*(1)	*E*(2)	νCO (cm^–1^)
**ReP**∩**N**	*E*_1/2_ = −1.75 V	*E*_pc_ = −2.78 V	2023, 1928, 1896
**ReAs**∩**N**	*E*_1/2_ = −1.81 V	*E*_pc_ = −3.22 V	2023, 1925, 1897
**Re1**	*E*_1/2_ = −1.84 V	*E*_pc_ = −2.34 V	2019, 1913, 1891
**Re2**	*E*_1/2_ = −1.69 V	*E*_pc_ = −2.34 V	2017, 1910, 1888
**ReP**∩**N**^•–^(A)			2005, 1906, 1865 (b)
**ReP**∩**N**^•–^(B)			1999, 1895, 1863 (a)
**ReP**∩**N**^•–^(H^+^)			1998, 1892, 1864 (a)
**ReAs**∩**N**^•–^(A)			2004, 1902, 1870 (b)
**ReAs**∩**N**^•–^(B)			2000, 1893, 1864 (a)
**ReAs**∩**N**^•–^(H^+^)			1999, 1891, 1866 (a)

The trIR data provide lower resolution for the spectra compared to the FT-IR-SEC data due to the use of either a 64 or 32 pixel MCT detector. At the same time, only difference spectra are obtained.

### Time-Resolved Infrared Spectroscopy

Standard reductive quenching was used to photochemically produce reduced **ReP**∩**N**. We took advantage of the fact that **ReP**∩**N** and **ReAs**∩**N** show a high quantum yield for photoreduction because their room-temperature excited-state lifetime is up to 10 times longer than that of the Re-diimine complexes. Photochemically formed **ReP**∩**N**^•–^ should react in the same way as electrochemically prepared **ReP**∩**N**^•–^, if the changes in solvent, electrolytes, and sacrificial electron donors do not interfere. We thus need an electron donor which does not absorb in the visible range and does not easily coordinate to Re. The sacrificial electron donor molecule 1,3-dimethyl-2-phenylbenzimidazoline (BIH)^29^ was prepared following a published procedure^30^ and used in combination with DBU (1,8-diazabicyclo[5.4.0]undec-7-en) as a non-nucleophilic base.^31^ DBU can deprotonate the BIH^•+^ radical cation more efficiently (at much lower concentration) than commonly used TEOA (triethanolamine) and increase the yield of reduced **ReP**∩**N**^•–^ by a second electron transfer to ground state **ReP**∩**N**, while also limiting electron back transfer to BIH^•+^ from **ReP**∩**N**^•–^.^30,31^ To initiate the photoreaction, we excited the charge transfer bands of **ReP**∩**N** and **ReAs**∩**N** and recorded transient infrared spectra in the carbonyl stretch region over 10 orders of magnitude in time. “Wet” CO_2_ was used as a proton source.

**Figure 7:**
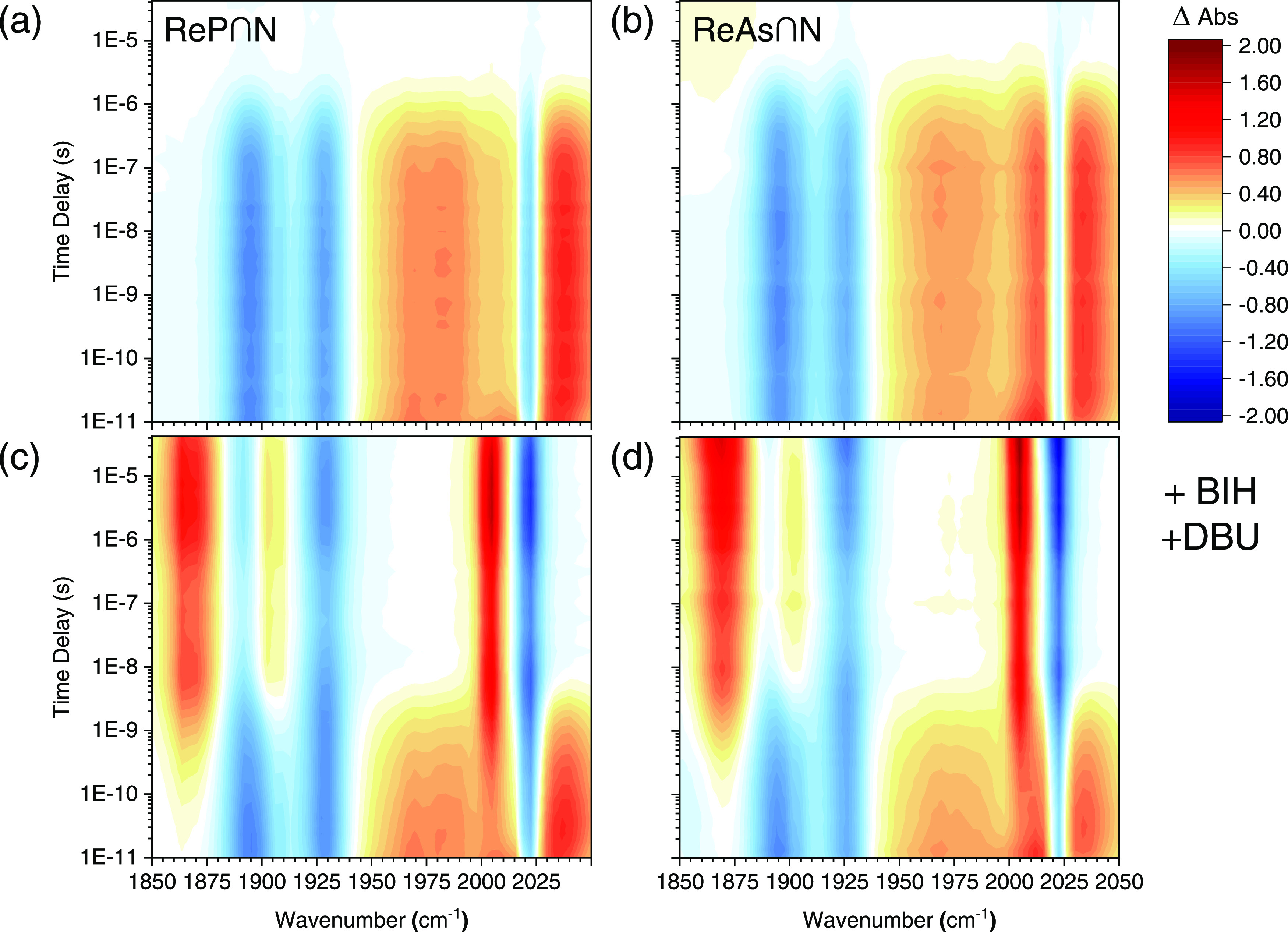
Contour plots of time-resolved IR measurements of **ReP**∩**N** (a) and **ReAs**∩**N** (b) in Ar-purged DMSO (top) and with 100 mM BIH and 30 mM DBU (c, d). Positive signals, which include excited-state absorption and reduced **ReP**∩**N** are shown in red, and negative signals from the ground state bleach are depicted in blue. The additional bleach near 1860 cm^–1^ is due to photoproduct accumulation in the sample after approximately 20 min of measurement.

Figure 7a shows contour plots of the early stages of the photoreaction in the absence of quencher and “wet” CO_2_. The triplet excited state of **ReP**∩**N** can be identified by the increased absorption (red) of the ν_CO_ at 2040, 1990, and 1965 cm^–1^. The depleted ground state can be seen as a negative signal (blue) at 2023, 1928, and 1896 cm^–1^ for the a′(1), a″, and a′(2) ν_CO_ modes, respectively. The decay of the signal reflects the very long excited-state lifetime (≈1 μs) and the slightly longer excited lifetime of **ReAs**∩**N** compared to **ReP**∩**N** also in DMSO. Figure 7c,d shows the same measurements in the presence of 0.1 M BIH and 30 mM DBU as a sacrificial electron donor. The triplet state signals are much shorter lived, and new positive absorption bands appear at 1998, 1910, and 1862 cm^–1^ as a result of the formation of singly reduced **ReP**∩**N**^•–^. On a 100 ns time scale both the positive **ReP**∩**N**^•–^ signal as well as the negative ground state bleach signal become slightly smaller, indicating geminate recombination of the reduced **ReP**∩**N**^•–^ with the BIH^•+^ radical cation. In the absence of DBU (see Figure S12b), this effect is quite pronounced: while the quenching efficiency is nearly 100%, thanks to the long excited-state lifetime of the complex, only approximately half of the initially excited molecules would remain permanently reduced after 10 μs in the absence of the base. In Figure 7c,d, one can see that upon further addition of DBU as a base, both ground state bleach of **ReP**∩**N** as well as the positive band of **ReP**∩**N**^•–^ are increasing again on a 1–10 μs time scale. This is the result of direct electron transfer to ground state **ReP**∩**N** from BI^•^, which was formed via deprotonation of BIH^•+^,^30,31^ thus significantly increasing the yield of **ReP**∩**N**^•–^ for the investigation of its reactivity. However, in the time window covered by the trIR measurement shown in Figure 7 (up to 40 μs), the addition of CO_2_ did not result in significant changes of the signal.

**Figure 8:**
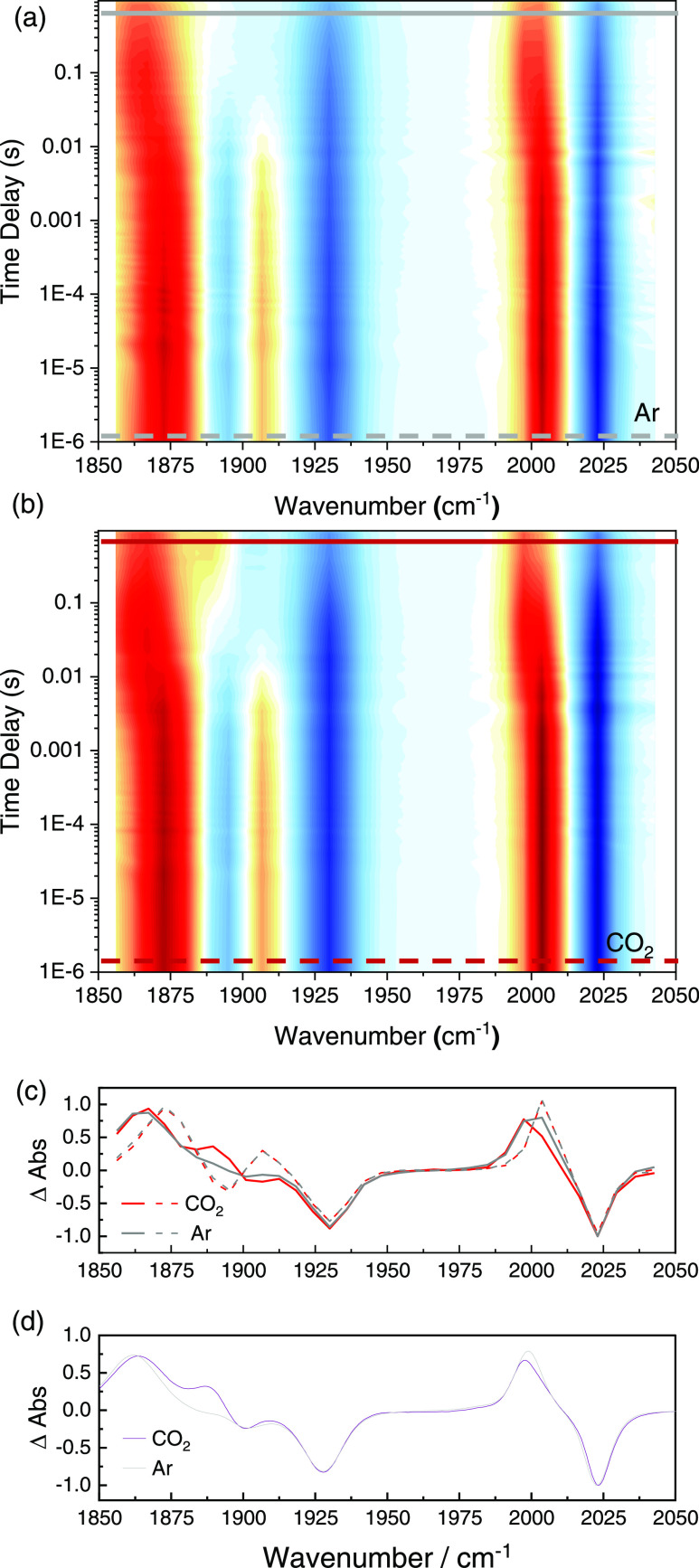
Normalized spectral transients of the time-resolved IR measurements of **ReP**∩**N** with 100 mM BIH as a sacrificial electron donor, 30 mM DBU as a base (a) with Ar (b) with “wet” CO_2_ (c) Transient cuts of the two spectra above at 1 μs (dashed) and at 800 ms (full lines). (d) SEC traces at −1.6 V for **ReP**∩**N** in DMSO with (violet) and without (gray) “wet” CO_2_.

Figure 8 compares the transient IR-data on time scales up to 1 s of **ReP**∩**N** with 100 mM BIH and 30 mM DBU under Ar (a) and in the presence of CO_2_ (b). Ten milliseconds after excitation, significant changes occur in both cases. The positive band at 1900 cm^–1^ disappears as all carbonyl stretch bands undergo a shift to lower frequency. This is a clear indication of slightly increasing electron density at the metal center due to the formation of a new species, presumably the product of the EC_rev_ mechanism. In the presence of protons from “wet” CO_2_, the situation is slightly different: an additional positive signal can be seen at 1875 cm^–1^, in analogy to the differences observed by spectroelectrochemistry (Figure 4d). Additionally, the positive signal around 2003 cm^–1^ also shifts further to a lower energy than in the absence of protons.

## Discussion

Our initial expectation for the reaction of **ReP**∩**N** (and **ReAs**∩**N**) after single-electron reduction was a similar behavior to **Re1**. The first reductive CV wave (**Re1**/**Re1**^–^) appears reversible at a variety of scan rates (see Figure S3(b)) aside from an (expected) increase of the peak separation for *v* > 50 mV s^–1^. *E*_1/2_ of **Re1**/**Re1**^–^ is always clearly observable and does not change. However, this is not the case for **ReP**∩**N**^–^. Although the first reductive CV wave is clearly due to single-electron transfer, it is strongly and surprisingly inversely scan rate-dependent. A possible scheme explaining this observation is an EC_rev_ mechanism with a reversible chemical step as shown in eq 1.
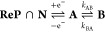
1

**ReP**∩**N** turns into **A** upon single-electron reduction, which is in equilibrium with another species **B**. The equilibrium is strongly on the side of **B**, the dominant single-electron reduction product, which is oxidized at −0.8 V vs Fc/Fc^+^, while **A** is oxidized back directly to the parent **ReP**∩**N** at a lower potential. At slow scan rates (all) singly reduced **ReP**∩**N**^–^ can be oxidized back at this lower potential because the equilibrium between A ⇌ B is re-established fast enough.

The forward rate *k*_AB_ from **A** to **B** can be deduced from the trIR measurements. **A** is formed on a nanosecond time scale by diffusion-controlled reductive quenching. The 100 ms spectrum is identical with the dominant reduction product in the FT-IR-SEC experiment, which we assign to **B**. It is formed at a rate of 200 s^–1^. Knowing this forward rate, the rate for back reaction *k*_BA_ can be estimated through kinetic analysis of the cyclic voltammogram^32^ yielding ≈6 s^–1^ (see the SI, Figure S14).

There are a few possibilities that can explain this electrochemical behavior, some of which can be supported by additional experiments. The first idea would be the unusual loss of Cl^–^ after a single-electron transfer. **B** would then be actually two species, **ReP**∩**N**^•^ + Cl^–^. This would make the CV wave irreversible and could also explain the back oxidation of a **ReP**∩**N**^•^ radical or the solvated adduct of this radical at a higher potential than the initial **ReP**∩**N**/**ReP**∩**N**^•–^ redox pair, as this species then would carry no negative charge. The oxidative wave at higher potential is not reversible, and no corresponding cathodic peak can be observed. This could be explained by fast back addition of Cl^–^ to the formed **ReP**∩**N**^**+**^ or ligand (solv) exchange in the formed **ReP**∩**N(solv)**^**+**^ species. However, the recovery of the initial complex would be a bimolecular process and slow compared to the time scale of the CV experiment. In addition, large excess of Cl^–^ (100 mM Cl^–^ vs 5 mM **ReP**∩**N**) did not change the CV (see Figure S5).

If there is no loss of Cl^–^—and no release of CO since the typical pattern for a *fac*-tricarbonyl IR mode is preserved—other possibilities are the opening of the Re–P or the Re–N bonds. The former is unlikely as the time scales for the formation of **B** in the transient IR-data is very similar for **ReP**∩**N** and **ReAs**∩**N**. It is also sterically more difficult than a simple rotation around the Re–P bond, i.e., the alternative opening of the Re–N bond. The latter could lead to a reversible isomerization of the quinoline moiety, which would be entropically favored. Indeed, the DFT calculations indicate weakening of the Re–N bond upon reduction. However, the same calculations are difficult to converge for the open form and strongly overestimate the red shift of the carbonyl modes. Any mechanism should provide an explanation for the observed perturbation of the **A** ⇌ **B** equilibrium in the presence of an acid:
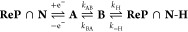
2

In the presence of “wet” CO_2_, there is a more pronounced second reductive peak at −2.3 V vs Fc/Fc^+^ (Figure 2a), possibly due to a protonated and hence neutral compound **ReP**∩**N**^•^-**H** that is easier to reduce than **ReP**∩**N**^•–^.

We thus need to address the question of where a possible protonation could take place, which leads to very large changes in the UV–vis absorption but only to small changes in the carbonyl stretch modes. Attempts to analyze the singly reduced, protonated species by NMR failed, likely due to its radical character. Further reduction to the double reduced protonated species (which would then ideally not be a radical) did not proceed clean enough for an NMR analysis either. We suspect, that the double reduced protonated species is not stable enough. DFT calculations of the singly reduced species suggest that one of the SOMOs results in an antibonding interaction between the Re center and the N atom of the quinoline ligand (see Figure S15). If the Re–N bond is weakened, H^+^ might be able to add to the chelating ligand (e.g., at the N position), which is expected to be very basic once reduced, or H^+^ addition may lead to the protonation of the reduced aromatic system—a reactivity which is not unprecedented as quinoline has a quite rich redox chemistry.^33^ Both should have only a small effect on the CO-stretch bands.

**Scheme 3:**
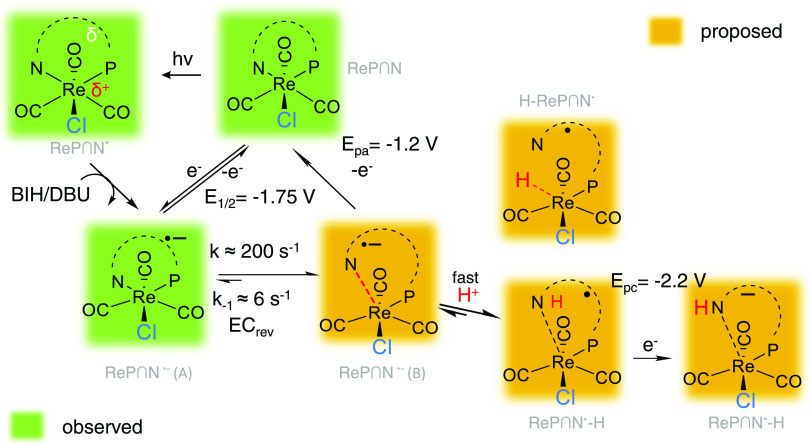
Proposed Reaction Pathways of **ReP**∩**N** upon Reduction, with and without Water/Protons

Alternatively, the proton could bind to the metal, forming a rhenium hydride (**H**-**ReP**∩**N**^•^). However, this is expected to cause a more significant perturbation of the carbonyl stretch spectrum. Also, if sufficient amount of hydride is present, a (bimolecular) process involving **H**-**ReP**∩**N** could potentially lead to H_2_ formation. If this process is fast enough, a catalytic wave should be observed with a strong acid, such as trifluoroacetic acid (TFA). However, the observed increase in current is not very large compared to the direct reduction of protons (see Figure S7). We thus suggest that reversible protonation takes place on the quinoline moiety.

(Scheme 3) summarizes the mechanism derived from the combination of electrochemical, spectroelectrochemical, and trIR data. The top of this figure depicts two ways to obtain the singly reduced **ReP**∩**N**^•–^. Electrochemical reduction at *E* < −1.75 V vs Fc/Fc^+^ (left side) or photochemical reduction with the BIH/DBU system (right side) both lead to the formation of singly reduced **ReP**∩**N**^•–^. This intermediate can be observed best in the trIR data at delays <10 ms (dashed lines in Figure 8c). From trIR spectroscopy, we see that the reduced **ReP**∩**N**^•–^ is lost with a rate of approximately 200 s^–1^ both with and without additional protons. Hence, this step is rate-limiting for any follow up reaction.

The trIR traces at 800 ms delay and the SEC difference spectra are quite similar (Figure 8). This means that at the end of the trIR experiment, i.e., within one second after (photo)reduction of **ReP**∩**N**, the system has already largely equilibrated and mostly protonated complexes are present. A time scale of this order is already implied by the scan rate dependence of the first reduction peak in the CV data (Figure 3), which is partially irreversible already for a scan time of less than one second in the presence of “wet” CO_2_. In this respect, all three experiments provide consistent results, but only the combination of all three allowed us to draw a detailed mechanistic picture.

We would like to note that we initially favored a mechanism of Cl^–^ release and CO_2_ binding to the metal because in the SEC measurements under CO_2_ bubbling, the growth and decay of the bicarbonate bands at 1669/1623 cm^–1^ occur in parallel to the spectral shifts in the carbonyl stretch region. In retrospect, this can be explained by the shift in pH through the protonation of the radical cation, which strongly influences the bicarbonate H_2_CO_3_ equilibrium .

Finally, in analogy to **Re1** and **Re2**, **ReP**∩**N** and **ReAs**∩**N** can also be doubly reduced at −2.75 V. Only at this potential, Cl^–^ is probably lost, and the addition of (dry) CO_2_ causes a comparably large current increase (see Figure 6, green curves). However, at this potential, there may already be a direct reduction of CO_2_.

## Conclusions

Many CO_2_ reduction systems address the question of CO_2_ reduction vs proton reduction, mainly because the reduction potential of CO_2_ is more negative than that of reduction of H_2_. Selectivity is a key feature for this catalysis. Initially, we considered that CO_2_ binds to the one-electron-reduced complex after losing its axial chloride ligand, which is the crucial step that ultimately renders this reaction irreversible. However, a deeper investigation leads us to a different and more detailed picture of the mechanism. In fact, as CV scans were repeated under strictly water-free conditions with CO_2_ dried over molecular sieves rather than taken directly from the cylinder, the presence of CO_2_ has no large effect on the kinetics (cf. Figure S6). A combined approach of spectroelectrochemistry (FT-IR and UV–vis) and transient IR spectroscopy helped us to understand this behavior. The latter elucidated the early time scales with the isomerization reaction that cannot be resolved in the spectroelectrochemical measurement. Based on these findings we have to infer that the novel complexes favor a proton-related reaction over CO_2_ reduction, although we have not investigated in detail whether CO_2_ could be reduced by a hydride transfer reaction of some sort of active species formed in our system under differing conditions. However, we cannot find any indications of a significant CO_2_-related redox chemistry in our experimental results. Therefore, the presented molecules could be regarded as a very costly H^+^ plus e^–^ “storage” system but surely much cheaper and more efficient systems exist, for example, quinone-type molecules.^34^ Interestingly, from the observation that there is an increase in current after the second reduction of **ReP**∩**N** and **ReAs**∩**N** in the presence of CO_2_, there are indications for a similar behavior to **Re1** and **Re2** (i.e., axial ligand release, CO_2_ binding/reduction, etc.). Normally, the presence of protons facilitates the CO_2_ reduction process.^35^ However, the catalysts have to be well chosen to obtain optimal performance and selectivity toward CO_2_ reduction. With the available data, it is not clear whether **ReP**∩**N** and **ReAs**∩**N** fulfill these requirements and reduce CO_2_ over H^+^, which would be subject of further studies.

## Experimental Section

### General

8-(Diphenylphosphino)quinoline (P∩N) and 8-(diphenylarsino)quinoline (P∩N) were synthesized following a literature procedure.^36,37^ Complexation was done analogous to the synthesis of Lehn’s catalyst in toluene.^38,39^ Synthesis of BIH was done according to a published procedure,^30^ albeit on a smaller scale.

#### (P∩N)Re(CO)_3_Cl, ReP∩N

The complex was synthesized analogously to a previously reported procedure:^38^ P∩N (50 mg, 0.16 mmol) and Re(CO)_5_Cl (58 mg, 0.16 mmol) were refluxed in toluene (5 mL) for 2 h. After being cooled, the precipitated yellow crystals were filtered and dried in vacuo. Yield: 93 mg, (94%). ^1^H NMR (300 MHz, CDCl_3_): δ = 9.60 (dd, *J* = 5.0, 1.7 Hz, C2-H, 1 H), 8.45 (ddd, *J* = 8.5, 1.4 Hz, 1 H), 7.89–8.10 (m, 4 H), 7.76 (ddd, *J* = 8.8, 0.8 Hz, 1 H), 7.39–7.56 (m, 7 H), 7.28–7.38 (m, 2 H). ^13^C NMR (75.5 MHz, CDCl_3_): δ = 159.21 (d, *J*_CP_ = 4.4 Hz), 139.64 (d, *J*_CP_ = 39.8 Hz), 134.83 (d, *J*_CP_ = 11.1 Hz), 134.63 (s), 133.94 (s), 133.25 (s), 132.67 (s), 132.26 (s), 132.11 (s), 131.32 (s), 130.59 (d, *J*_CP_ = 2.2 Hz), 130.39 (d, *J*_CP_ = 8.9 Hz), 128.9 (d, *J*_CP_ = 6.6 Hz), 128.74 (d, *J*_CP_ = 7.4 Hz), 128.21 (d, *J*_CP_ = 12.5 Hz), 128.05 (s), 123.49 (s). ^31^P NMR (121.49 MHz, CDCl_3_): δ = 23.70 ppm. MS (EI, 70 eV): *m*/*z* (%) = 618.8 (16) [M]^+^, 590.9 (87) [M – CO]^+^, 562.9 (51) [M – 2CO]^+^, 534.9 (74) [M – 3CO]^+^, 380.8 (100) [qnClPRe]^+^, 204.1 (51) [C_15_H_10_N]^+^. C_24_H_16_NPReO_3_Cl (619.02 g/mol): calcd C 46.57, H 2.61, N 2.26; found C 46.60, H 2.68, N 2.24.

#### (As∩N)Re(CO)_3_Cl, ReAs∩N

The complex was prepared as described for the phosphorus congener: As∩N (100 mg, 0.28 mmol) and Re(CO)_5_Cl (101 mg, 0.28 mmol) were refluxed in toluene (10 mL) for 2 h. After cooling, the precipitated yellow crystals were filtered and dried in vacuo. Yield: 161 mg, (87%). Analytical data: MW = 662.97 g/mol. ^1^H NMR (300 MHz, CDCl_3_): δ = 9.73 (dd, ^3^*J*_HH_ = 5.1 Hz, ^4^*J*_HH_ = 1.5 Hz, 1 H), 8.47 (dd, ^3^*J*_HH_ = 8.2 Hz, ^4^*J*_HH_ = 1.7 Hz, 1 H), 8.30–8.50 (m, 1 H), 8.01 (d, ^4^*J*_HH_ = 0.6 Hz, 1 H) 7.79 (dd, ^3^*J*_HH_ = 7.8 Hz, ^4^*J*_HH_ = 1.8 Hz, 1 H), 7.74 (pseudo-t, ^3^*J*_HH_ = 7.5 Hz, 1 H), 7.53 (dd, ^3^*J*_HH_ = 8.2 Hz, ^3^*J*_HH_ = 5.2 Hz, 1 H), 7.40–7.49 (m, Ph-H, 8 H). ^13^C NMR (75.5 MHz, CDCl_3_): δ = 159.92 (C2), 150.80, 141.78, 140.50, 138.67, 137.95, 134.14 (Ph), 133.78, 132.31 (Ph), 132.12, 130.63, 130.60, 130.34, 129.37 (Ph), 129.21 (Ph), 128.18, 123.30. MS (EI, 70 eV): *m*/*z* (%) = 662.8 (14) [M]^+^, 634.7 (50) [M – CO]^+^, 424.8 (100) [AsqnReCl]^+^, 357.0 (38) [Ph_2_Asqn]^+^, 279.9 (26) [PhAsqn]^+^, 204.0 (61) [C_15_H_10_N]^+^, 151.9 (9) [PhAs]^+^. C_24_H_16_NAsReO_3_Cl (662.97 g/mol): calcd C 43.48, H 2.43, N 2.11; found C 43.86, H 2.62, N 2.01.

### Crystal Structure Determination

Single-crystal X-ray data of the rhenium complexes were collected on a Stoe-IPDS diffractometer.^40^ The diffractometer uses graphite-monochromated Mo Kα radiation (λ = 0.71073 Å). The crystal structures were determined by direct methods (SIR-97^41^), followed by full-matrix least-squares refinements on F^2^ (Olex2^42^). The hydrogen atoms were calculated geometrically, and a riding model was applied during the refinement process.

### Spectroscopy

UV–vis spectra were recorded with a Shimadzu UV-2450 spectrometer, and FT-IR spectra were recorded with a Bruker Tensor 27.

Samples for trIR containing 5 mM **ReP**∩**N**, 100 mM BIH, and ≈30 mM DBU were purged with Ar/CO_2_ > 20 min prior to each measurement. Time scales from 30 ps to 40 μs were measured with a system of two electronically synchronized Ti–sapphire laser systems operating at a repetition rate of 2.5 kHz.^43^ One laser was used to produce the mid-IR probe pulses from the 780 nm fundamental by optical parametric amplification followed by difference-frequency mixing.^44^ The 420 nm pump pulses were generated by the frequency doubling of the 840 nm light from the second Ti–sapphire laser. Longer time scales up to 1 s were covered with another setup. Pump pulses at 1 Hz were generated with a Q-switched nanosecond laser (CrystaLaser PL-2003 at 447 nm). For each excitation, a sequence of 100,000 mid-IR pulses at 100 kHz from a fiber amplifier (Amplitude) and OPA (Fastlite) were used to probe the reaction.^45^

### Electrochemistry and Spectroelectrochemistry

The typical solvents for electrochemical studies of Re tricarbonyls are acetonitrile, THF, and DMF.^3,4^ We used DMF for the CV experiments. For spectroelectrochemistry and transient IR spectroscopy, however, we used DMSO because of better solvent transparency in the 1500–2300 cm^–1^ region. The reactivity of rhenium diimines has been observed to be similar in both solvents;^46^ however, CO_2_ solubility is slightly better in DMF (0.194 ± 0.014 M) than in DMSO (0.131 ± 0.007 M at 25 °C).^47^

We found it necessary to monitor the amount of water in the solution. Generally using a nitrogen-filled flow-box for most of the sample preparation and handling except the CO_2_ purging and commercially available dry solvents, we can estimate the amount of water in the solvent by its IR absorbance in the 3000 and 1600 cm^–1^ ranges. We repeatedly observed a significantly larger amount of water in the samples after bubbling with CO_2_, even though the gas stream (CO_2_ 4.5) had only 5 ppm water.

Estimation of the water content in the samples could also be done from the FT-IR-SEC data: 1 μm of pure liquid water (55 M) gives rise to a 0.55 OD peak absorption in the OH-stretch region.^48^ After bubbling “wet” CO_2_ 4.5 for only a few minutes in DMSO, we observe roughly 0.05 OD in a 200 μm cell, which corresponds to a water concentration of ≈55/200/10 = 27 mM.

Cyclic voltammetry of all complexes (at 5.0 ± 0.2 mM concentration of analyte if not otherwise stated) was performed in DMF containing 0.1 M TBAPF_6_ as a conducting electrolyte on a glassy carbon working electrode. The counter electrode used was made of Pt, and the potential was applied against a Ag quasi-reference electrode separated from the main solution by a porous glass frit. The applied potential was calibrated internally against the Fc/Fc^+^ redox pair. Performing the full experiment in a nitrogen-purged glovebox instead of using an Ar blanket gas approach on the lab bench combined with attempts to predry the CO_2_ gas from the cylinder over molecular sieve (3 Å) as well as limiting the sparging time of the introduced CO_2_ to around 5 min thus leads to a decrease of the second reduction peak, indicating that the presence of water in the CO_2_ plays a major role. Spectroelectrochemistry (SEC) was performed in dry DMSO (0.3 M TBAPF_6_) with an OTTLE cell equipped with CaF_2_ windows (IR) and Pt grid working as a counter electrode as well as a Ag wire as a quasi-reference electrode.^4,49^ The applied potential was calibrated externally against the Fc/Fc^+^ redox pair. Two different SEC methods were applied. Either many spectra were recorded during the scanning of a CV at 2 mV s^–1^ (CV mode) or only a few steps to selected potential were applied and held for a few minutes until the current was somewhat stable (controlled potential electrolysis (CPE) mode). A series of FT-IR spectra were recorded to follow the reaction. We found that in the CV mode, the conversion of **ReP**∩**N** was far more complete than with the CPE methods. The reason could be the accuracy of the applied potential in the latter case and the fact that we could better adjust how far we scan with the CV method. However, in the presence of “wet” CO_2_, the conversion of **ReP**∩**N** to **ReP**∩**N**^•–^ was incomplete under comparable conditions, despite higher charge density measured during the scan in the presence of CO_2_.

## Data Availability

Data for all relevant figures in the main manuscript and the SI has been uploaded to a data repository: DOI: 10.5281/zenodo.8406253.

## Supporting Information Available

Compiled DFT calculation results, additional electrochemical and spectroelectrochemical analyses, transient infrared spectra, simulated electrochemical data, original results of elemental analyses (EA) mass spectrometry (MS), ^1^H NMR, ^13^C NMR, and ^31^P NMR spectra (PDF)
