# Supplementary material for: Unexpected Redox Chemistry of P∩N- and As∩N-Rhenium(I) Tricarbonyl Complexes in the Presence of CO2 Acting as an Acid
Source: Inorg Chem. 2023 Oct 6;62(42):17510–21. doi: 10.1021/acs.inorgchem.3c02925 (PMC10598882; doi:10.1021/acs.inorgchem.3c02925)
Supplement: Supplementary file 1 — ic3c02925_si_001.pdf [file ic3c02925_si_001.pdf]

Supporting Information: Unexpected Redox Chemistry of P∩N- and As∩N- Rhenium(I) tricarbonyl Complexes in Presence of CO_2 Acting as an Acid

Martin Ertl,† Uwe Monkowius,† and Kerstin T. Oppelt*,‡

†*Linz School of Education - Chemistry, Johannes Kepler University Linz, Altenberger Strasse 69, 4040 Linz, Austria*

‡*Department of Chemistry, University of Zürich, Winterthurerstrasse 190, 8057 Zürich, Switzerland*

E-mail: kerstin.oppelt@uzh.ch

Readme

This document provides additional experimental data to support the discussion main manuscript. The content is provided in parallel to the "Results" and "Discussion" section of the main manuscript for easier reference. Data for all relevant figures in the main manuscript and the SI has been uploaded to a data repository: DOI: 10.5281/zenodo.8406253.

Results

Photophysical Properties

The basic photophysical characterization of **Re$P\cap N$** and **Re$As\cap N$** was supplemented with QC calculations (Gaussian 16W v.1.1/DFT/B3LYP/6-31G(d,p)/LANL2DZ). Selected results about the frontier orbital energies and localization are shown below in Fig. S1.

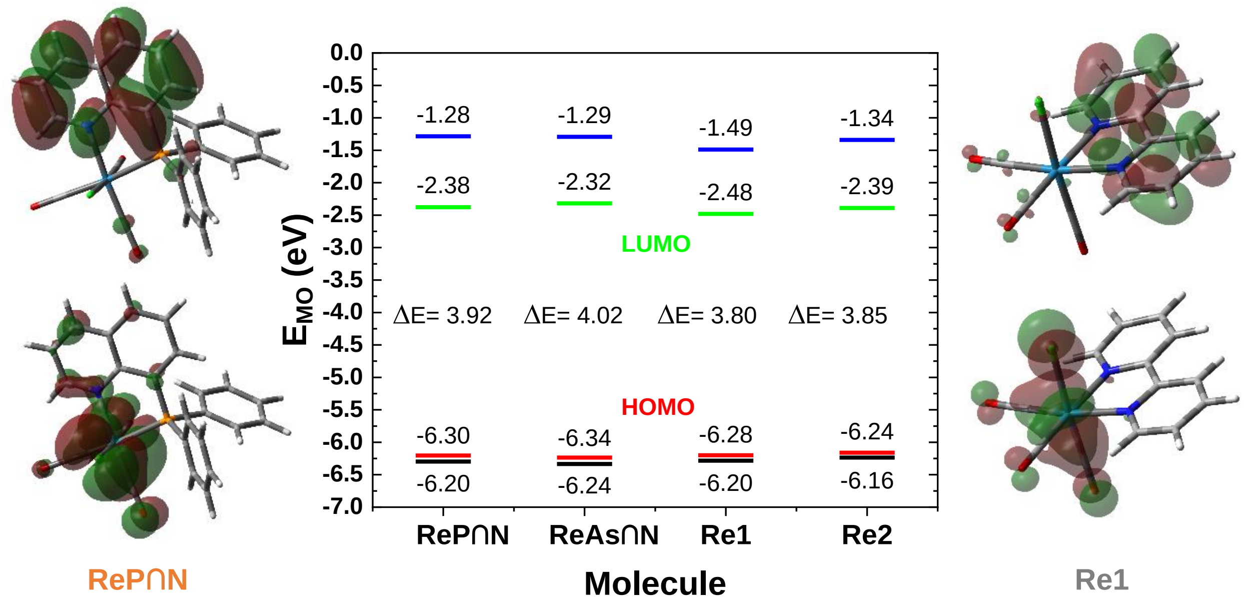

Figure S1: Calculated frontier orbital energies of **Re1**, **Re2**, **Re$P\cap N$** and **Re$As\cap N$**. HOMO and LUMO isosurfaces (at 0.02 Å) of **Re$P\cap N$** and **Re1** are depicted to the left and to the right.

It can be seen, that the HOMO-LUMO gap is larger for **Re$P\cap N$** and **Re$As\cap N$** than for **Re1** and **Re2**. The ligand centred LUMOs are at higher energy compared to **Re1** and **Re2** and the largely metal centred HOMOs are at slightly lower energy. The energy gap is also slightly larger for **Re$As\cap N$** compared to **Re$P\cap N$**, which is consistent with a shorter wavelength for the UV-vis absorption for **Re$As\cap N$**.

Electrochemistry and Spectroelectrochemistry

Reversibility of the first reduction can be seen well, when the peak current of the cyclic voltammetry (CV) waves are normalized to the reductive peak current such as can be seen in Fig. S2.

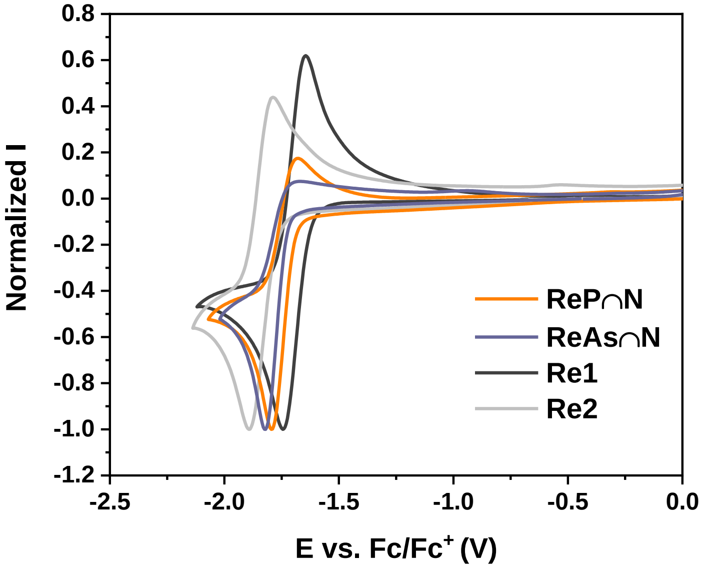

Figure S2: CV of **Re1**, **Re2**, **RePNN** and **ReAs∩N** in DMF/ 0.1 M $TBAPF_6$ in the potential range of the first (reversible) one electron reduction at 50 mV s^{-1}. The measured current density was normalized to the cathodic peak current.

In order to differentiate if the first reduction is a one electron process or involves the transfer of multiple charges at that potential, through an ECE mechanism for example, we compared the CVs of the first reduction of **Re1** and **RePNN** to the oxidation of ferrocene (Fc) at the same concentrations (5 mM) at different scan rates. The results are plotted in Fig. S3. The scans were normalized to the peak current of the ferrocene peak. Since the peak current of neither **Re1** nor **RePNN** change with the scan rate with respect to the peak current of Fc an ECE mechanism is unlikely and we think that for both **Re1** and **RePNN** the first reduction is a single electron transfer.

Fig. S4 shows variations of Cl^- concentration, different solvents and decrease in temperature of the solution as the first reductive wave was scanned at different scan rates to learn

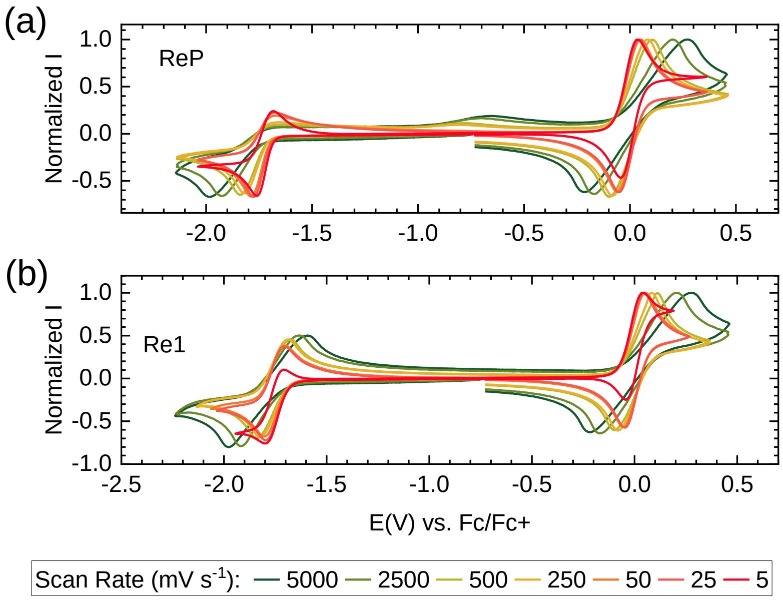

Figure S3: (a) Normalized CV of 5 mM **ReP∩N** in DMF/ 0.1 M $TBAPF_6$ in the potential range of the first (reversible) one electron reduction at scan rates from 5-5000 mV s^{-1} in comparison with 5 mM Ferrocene. The measured current density was normalized to the anodic peak of the ferrocene wave. (b) Normalized CV scans of 5 mM **Re1** under otherwise identical conditions.

more about possible chemical steps following the first reduction.

In order to make sure our trIR data measured in DMSO can be compared to the electrochemical measurements, we verified that the CV response of **ReP∩N** in DMSO is similar to the one in DMF, where most electrochemical measurements were done. The results are shown in Fig. S5.

When the reduction of **ReP∩N** was performed in the glovebox with pre-dried CO_2 instead of 'wet' CO_2 from the bottle, the increase of current at -2.2 V was much smaller, see Fig. S6.

Addition of low concentrations of a strong acid, trifluoroacetic acid (TFA), leads to a similar response as bubbling of 'wet' CO_2, see Fig. S7, however at higher concentrations of TFA the increasing cathodic wave at -2.2 V is overlapping with the strong current increase by direct proton reduction. This can be seen by comparison to reference data without the metal complex (scattered lines).

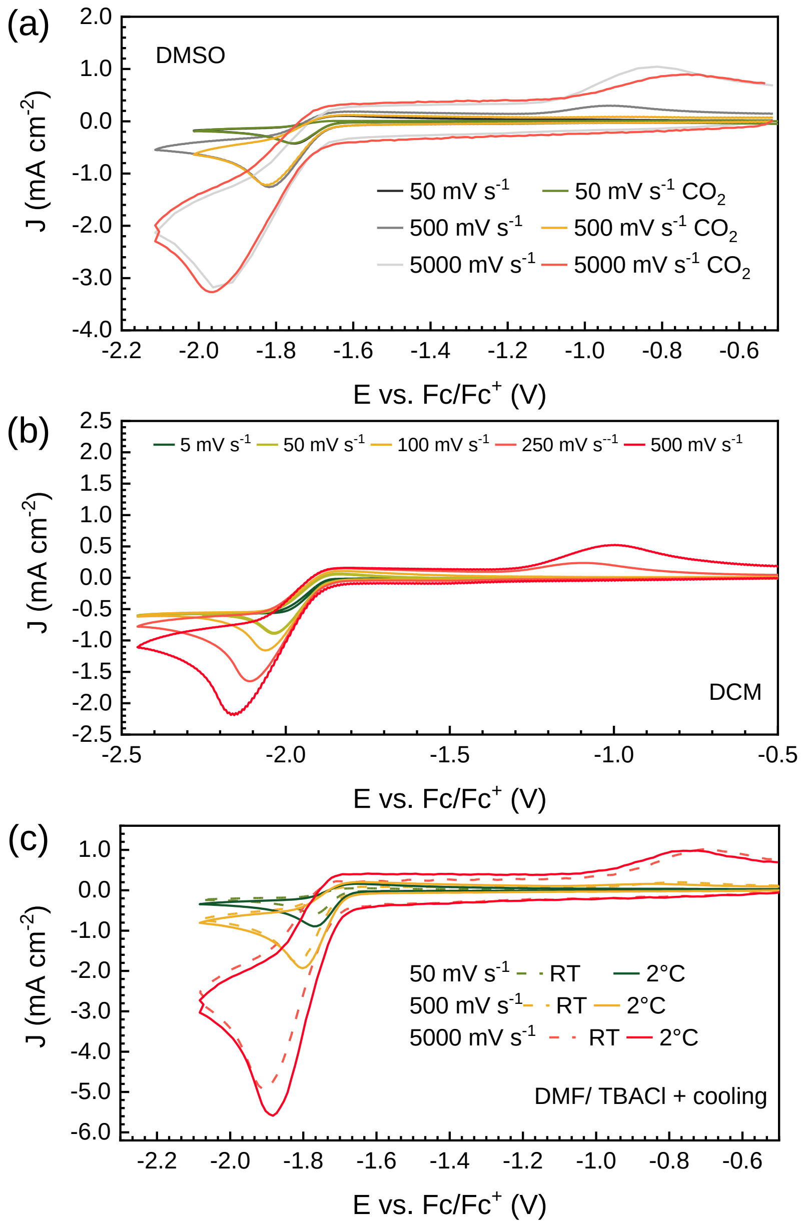

Figure S4: (a) Cyclic voltammetry of 5 mM **ReP∩N** in DMSO/ 0.1 M $TBAPF_6$ without CO_2 bubbling (grey lines) amd afer bubbling the solution with CO_2 4.5. (b) Cyclic voltammetry of 5 mM **ReP∩N** in 2°C cooled dichloromethane/ 0.1 M $TBAPF_6$ at a scan rates from 5-500 mV s^{-1}. (c) Cyclic voltammetry of 5 mM **ReP∩N** in DMF/ 0.1 M TBACl at a scan rates from 50-5000 mV s^{-1} at room temperature and at 2°C.

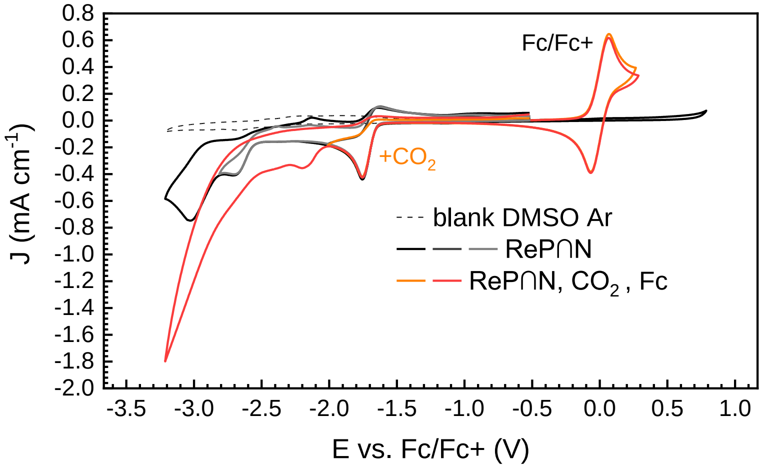

Figure S5: Cyclic voltammetry of 5 mM **RePਨN** in DMSO/0.1 M TBAPF$_6$ at a scan rate of 50 mV s^{-1}. The coloured scans were obtained after bubbling the sample solution with 'wet' CO_2 4.5 directly from the cylinder and addition of 5 mM ferrocene.

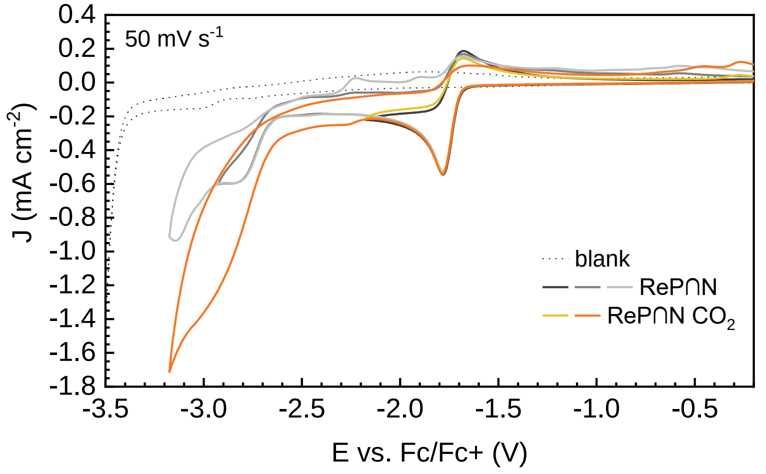

Figure S6: Cyclic voltammetry of 5 mM **RePਨN** in DMF/ 0.1 M TBAPF$_6$ at a scan rate of 50 mV s^{-1}. The coloured scans were obtained after bubbling the sample solution with pre-dried (molecular sieve, 3Å) CO_2.

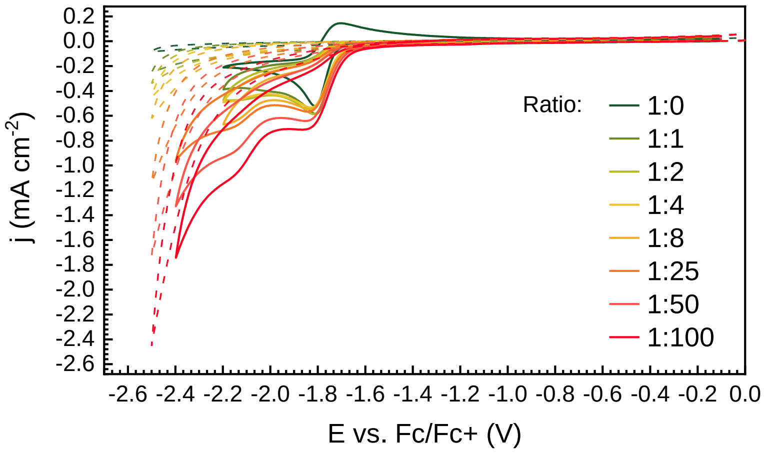

Figure S7: Cyclic voltammetry of 5 mM **ReP∩N** in DMF/ 0.1 M $TBAPF_6$ at a scan rate of 50 mV s^{-1} with increasing ratio of **ReP∩N**: trifluoroacetic acid. The dashed lines were measured without **ReP∩N** at the same concentrations of trifluoroacetic acid.

SEC was performed for **ReP∩N** and **ReAs∩N** in an OTTLE cell[1] with DMSO/ 0.3 M $TBAPF_6$ as conducting electrolyte. Samples were bubbled with either Ar or 'wet' CO_2 prior to scanning a CV at 2 mV s^{-1} while recording multiple FTIR spectra in equal intervals. Selected spectra are shown in Figs. S8 - S11.

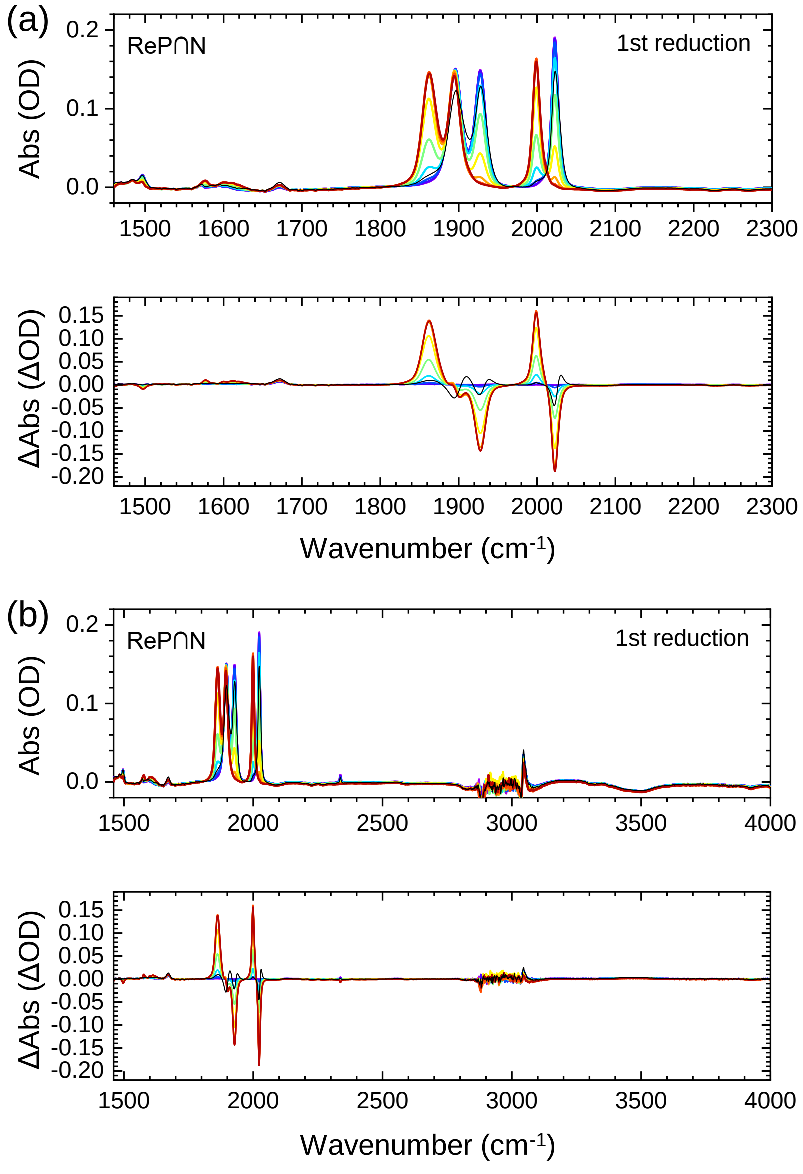

Figure S8: Selected FTIR-SEC transient spectra for **ReP∩N** under Ar recorded in CV mode at a scan rate of 2 mV s^{-1} (a) from 1450- 2300 cm^{-1} (b) from 1450- 4000 cm^{-1}.

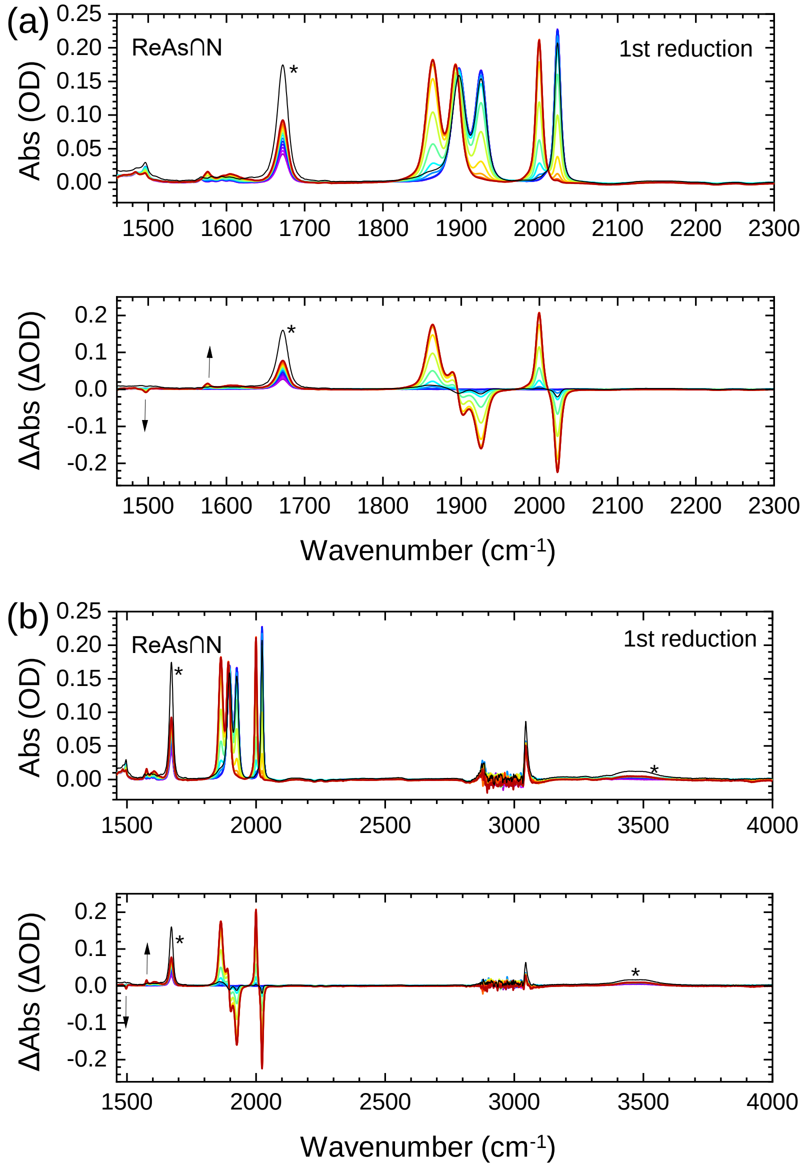

Figure S9: Selected FTIR-SEC transient spectra for **ReP∩N** with 'wet' CO_2 recorded in CV mode at a scan rate of 2 mV s^{-1}.The asterisk marks the signal of the changes in water absorbance (a) from 1450- 2300 cm^{-1}. (b) from 1450- 4000 cm^{-1}.

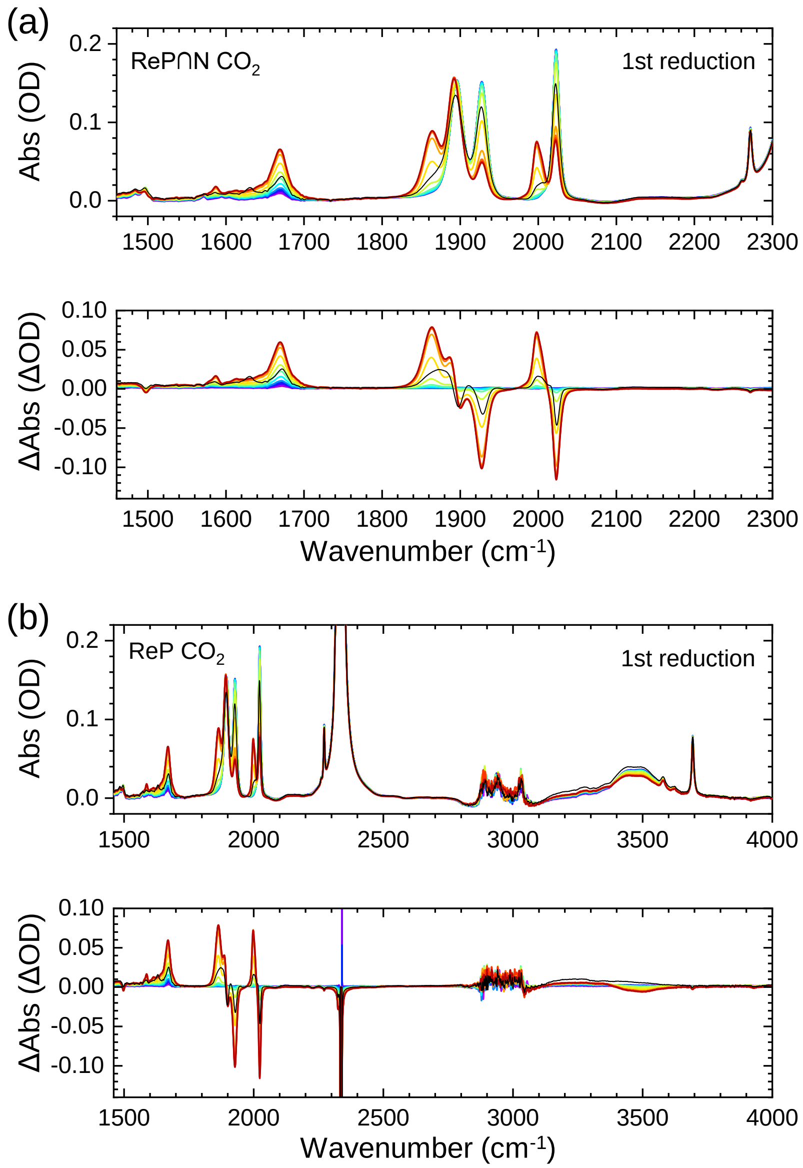

Figure S10: Selected FTIR-SEC transient spectra for **ReAs∩N** under Ar recorded in CV mode at a scan rate of 2 mV s^{-1}.(a) from 1450- 2300 cm^{-1} (b) from 1450- 4000 cm^{-1}.

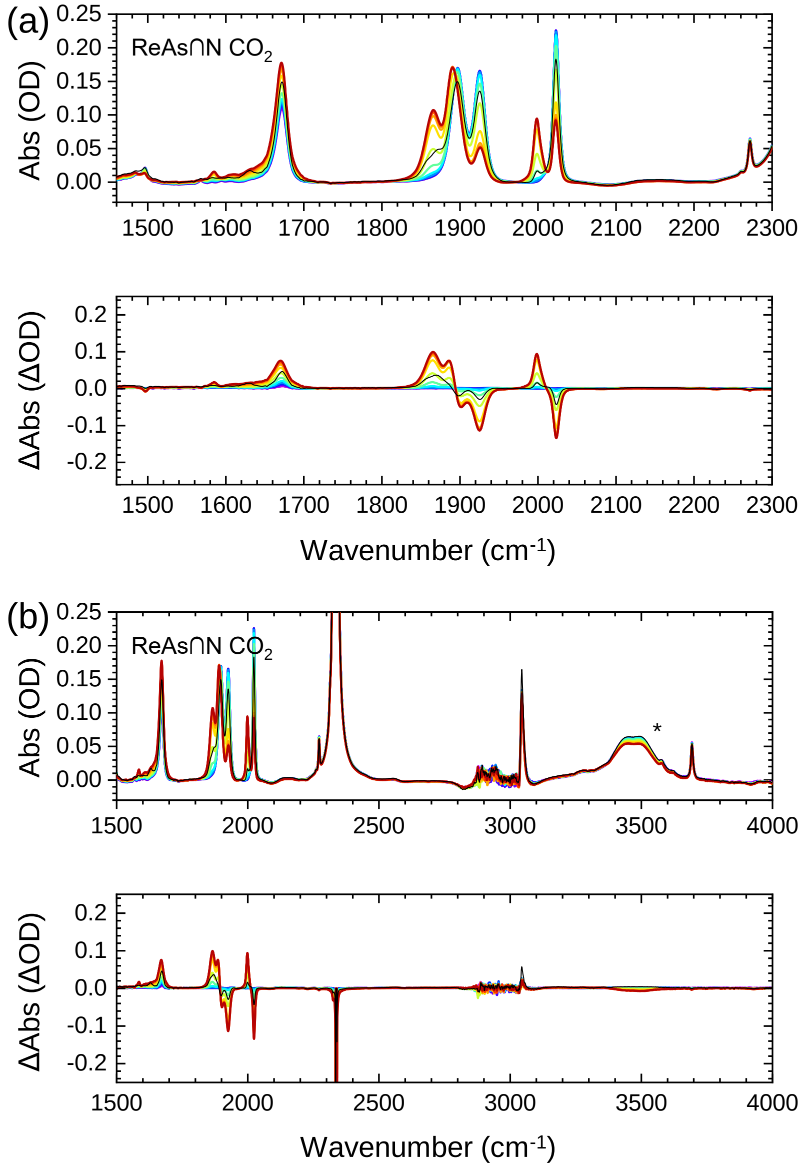

Figure S11: Selected FTIR-SEC transient spectra for **ReAs∩N** with 'wet' CO_2 recorded in CV mode at a scan rate of 2 mV s^{-1} (a) from 1450- 2300 cm^{-1} (b) from 1450- 4000 cm^{-1}.

Time Resolved Infrared Spectroscopy

Contour plots of the time resolved infrared spectroscopy in the carbonyl region of **ReP∩N** in DMSO under Ar are shown in Fig. S12(a)-(c). The negative (blue) signals is the ground state bleach. In Fig. S12(a) the positive (red) bands are exclusively the excited state absorption spectra of **ReP∩N** at higher frequency. In presence of BIH as a electron donor (Fig.S12(b)) these signals are shorter lived and new positive bands arise at lower frequency than the ground state bleach. However, the reduced **ReP∩N**$^{\cdot -}$ signal reaches a maximum around 10 ns and decays again towards longer delays due to geminate recombination. Addition of a non-nucleophilic base DBU can partly prevent geminate recombination, see Fig. S12(c), through deprotonation of the donor radical cation. This process forms a charge neutral and very reducing radical BI·.[2] It is in fact capable of reducing another **ReP∩N** molecule seen by an additional rise of the **ReP∩N**$^{\cdot -}$ signal at delays $>$500 ns in absence of any excited **ReP∩N*** molecule.

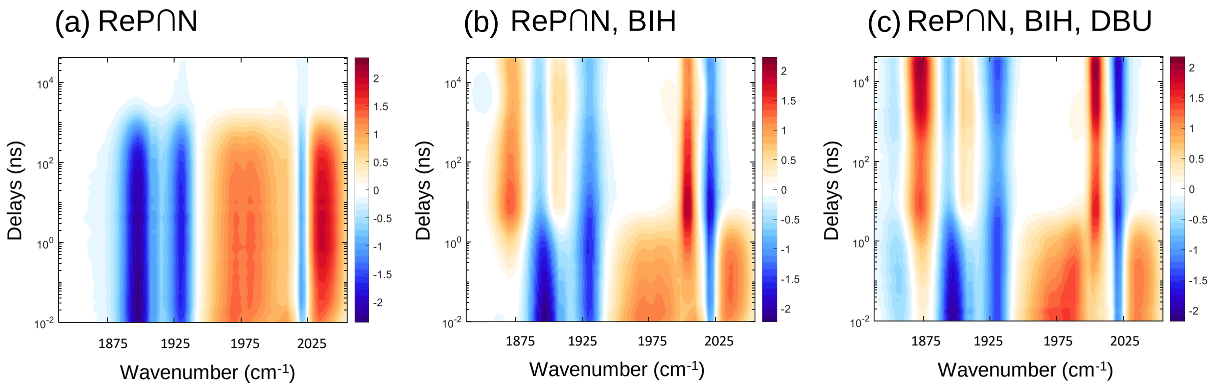

Figure S12: Time resolved infrared spectroscopy (trIR) on the ns timescale of **ReP∩N** (a) **ReP∩N** + BIH as a quencher (b) and **ReP∩N** + BIH and DBU as a non-nucleophilic base (c) Negative signals (ground state bleach) are blue, Positive bands (new species) are shown in red.

Figure S13 shows contour plots time resolved IR spectroscopy of **ReAs∩N** at long timescales. As in the other graphs, ground state bleach signals (negative) are depicted

in blue and excited state absorption as well the spectra of any newly formed species (positive bands) are shown in red. The difference upon addition of 'wet' CO_2 to the reduced **ReAs∩N** is almost invisible, compared to the case of **ReP∩N**. This is however in accordance to the smaller observed difference in the FTIR-SEC. It can be explained by the small shift in the energies of the tricarbonyl modes from **ReP∩N** to **ReAs∩N**. Nevertheless, a similar signal to the one we attributed to **ReP∩N**$^{\cdot-}$(A) is also visible here at times up to 10 ms, i.e. similar timescales as in the phosphine complex. Thus we propose formation of **ReAs∩N**$^{\cdot-}$(B) from **ReAs∩N**$^{\cdot-}$(A) in a similar fashion as proposed for **ReP∩N**.

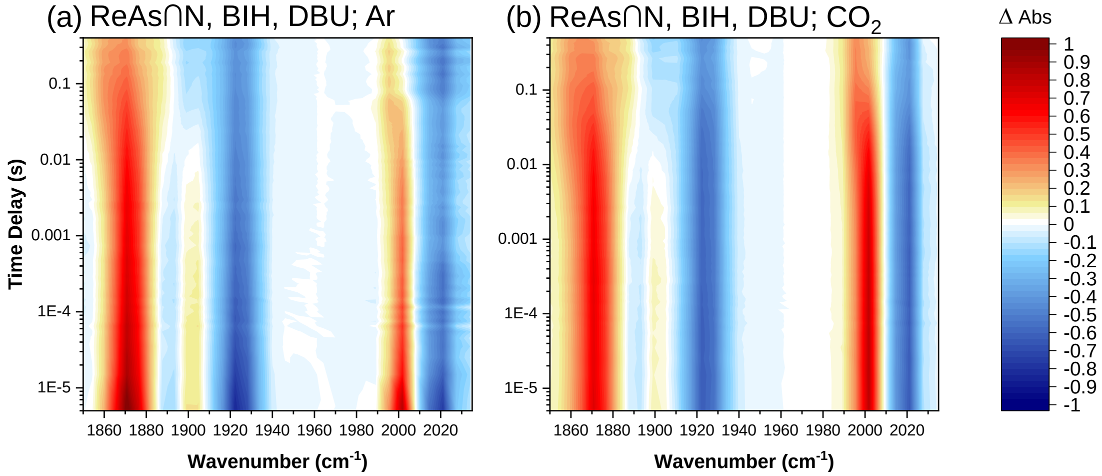

Figure S13: Normalized time resolved infrared spectroscopy (trIR) on the ns timescale of **ReAs∩N** (a) **ReP∩N** + BIH as a quencher and DBU as a non-nucleophilic base (b) The same sample after bubbling it with 'wet' CO_2. Negative signals (ground state bleach) are blue, Positive bands (new species) are shown in red.

Discussion

Scan rate dependent CV of **ReP∩N** were used to find the equilibrium constant for the isomerisation of **ReP∩N**$^{\cdot-}$**(B)**. The time constant for the observed changes in the trIR were used as a forward rate estimation to obtain the back reaction rate.

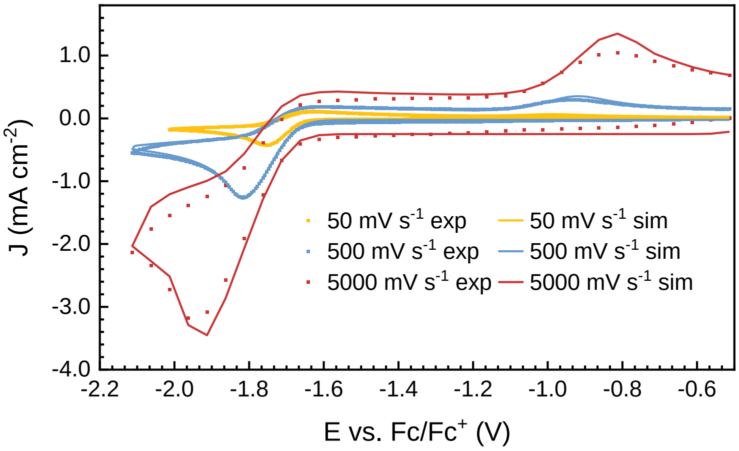

Figure S14: Simulated versus measured CV data for **Re$P\cap N$** at 50, 500 and 5000 mV s^{-1} in DMSO; k_f=200 s^{-1} (fix, from time resolved IR), $k_b\approx 6$ s^{-1} fitted; Estimated cell and diffusion parameters used for the fit: R_u= 35 Ω, double layer capacitance C_{dl}=50 µF, $D\approx 2\cdot 10^{-6}$ cm^2 s^{-1}.

$$
\begin{array}{ccc}
 & E_{1/2}(1) & \\
\mathbf{ReP\cap N} & \underset{-e^-}{\overset{+e^-}{\rightleftharpoons}} & \mathbf{ReP\cap N^{\cdot -}(A)} \\
fast \uparrow & & k_{BA} \upharpoonleft\downharpoonright k_{AB} \\
\mathbf{ReP\cap N(B)} & \underset{-e^-}{\overset{+e^-}{\rightleftharpoons}} & \mathbf{ReP\cap N^{\cdot -}(B)} \\
 & E_{ox}(B) &
\end{array}
\qquad \text{(S1)}
$$

The kinetic fitting model applied to the data shown in Fig.S14 shown in equation S1. A possible/plausible reaction from **Re$P\cap N$(B)** back to **Re$P\cap N$** was found to be fitted best if the reaction rate was assumed to be extremely fast »10000 s^{-1}. The forward rate k_{AB} was set to 200 s^{-1} to obtain a back reaction rate of around 6 s^{-1} for the isomerisation of **Re$P\cap N^{\cdot -}$(A)** to **Re$P\cap N^{\cdot -}$(B)**, thus $K_{eq} \approx 33$. The half wave potential for the first reversible reduction was fitted to be at -1.77 mV vs. Fc/Fc$^+$, close to the measured value at 50 mV s^{-1} as shown in Table 1 of the main text. The potential for the oxidation of **Re$P\cap N^{\cdot -}$(B)** was fitted to -0.81 mV.

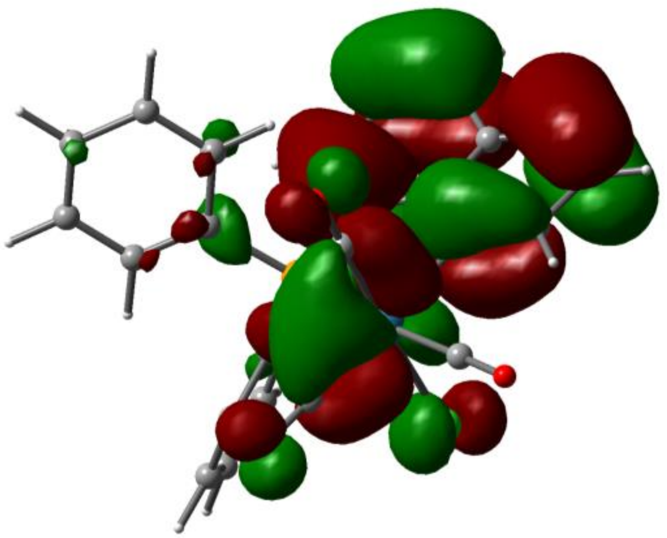

Figure S15: Calculated (Gaussian 16W v.1.1/DFT/uB3LYP/6-31G(d,p)/LANL2DZ) SOMO isosurface (at 0.02 Å) of **ReP∩N$^{\cdot-}$(A)**.

Experimental Section

Scans of the original elemental analysis data is provided here for further reference, see S16. We also include the MS data of **ReP∩N** (Fig.S17, S18) and **ReAs∩N** (S19, S20). Furthermore NMR spectra for both compounds with ^{31}P for **ReP∩N** is added for further reference.

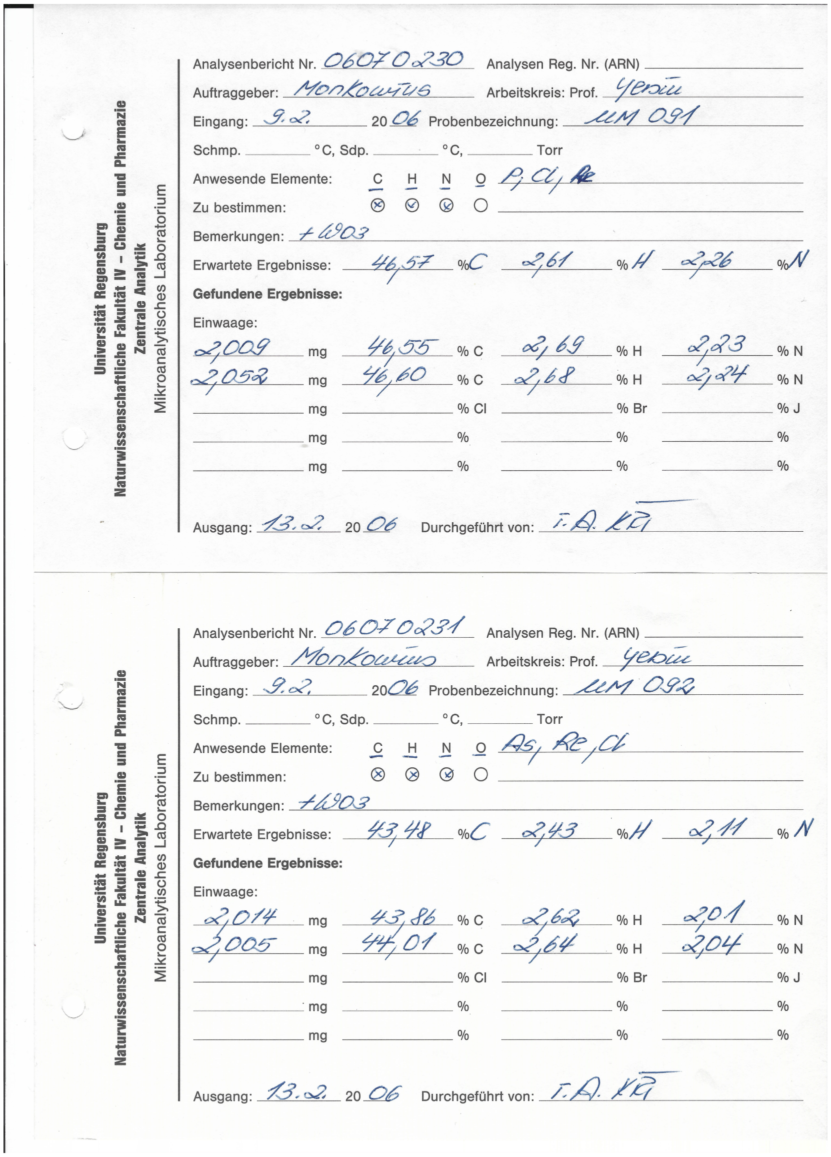
Universität Regensburg
Naturwissenschaftliche Fakultät IV – Chemie und Pharmazie
Zentrale Analytik
Mikroanalytisches Laboratorium

Analysenbericht Nr. 06070230 Analysen Reg. Nr. (ARN)
Auftraggeber: Monkowius Arbeitskreis: Prof. Yersin
Eingang: 9.2. 2006 Probenbezeichnung: UM 091
Schmp. °C, Sdp. °C, Torr
Anwesende Elemente: C H N O P, Cl, Re
Zu bestimmen: ⊗ ⊗ ⊗ ○
Bemerkungen: +WO3
Erwartete Ergebnisse: 46,57 %C 2,61 %H 2,26 %N
Gefundene Ergebnisse:
Einwaage:

2,009 mg	46,55	% C	2,69	% H	2,23	% N
2,052 mg	46,60	% C	2,68	% H	2,24	% N
mg		% Cl		% Br		% J
mg		%		%		%
mg		%		%		%

Ausgang: 13.2. 2006 Durchgeführt von: F.A. [illegible]

Universität Regensburg
Naturwissenschaftliche Fakultät IV – Chemie und Pharmazie
Zentrale Analytik
Mikroanalytisches Laboratorium

Analysenbericht Nr. 06070231 Analysen Reg. Nr. (ARN)
Auftraggeber: Monkowius Arbeitskreis: Prof. Yersin
Eingang: 9.2. 2006 Probenbezeichnung: UM 092
Schmp. °C, Sdp. °C, Torr
Anwesende Elemente: C H N O As, Re, Cl
Zu bestimmen: ⊗ ⊗ ⊗ ○
Bemerkungen: +WO3
Erwartete Ergebnisse: 43,48 %C 2,43 %H 2,11 %N
Gefundene Ergebnisse:
Einwaage:

2,014 mg	43,86	% C	2,62	% H	2,01	% N
2,005 mg	44,01	% C	2,64	% H	2,04	% N
mg		% Cl		% Br		% J
mg		%		%		%
mg		%		%		%

Ausgang: 13.2. 2006 Durchgeführt von: F.A. [illegible]

Figure S16: EA data for **ReP∩N** and **ReAs∩N**

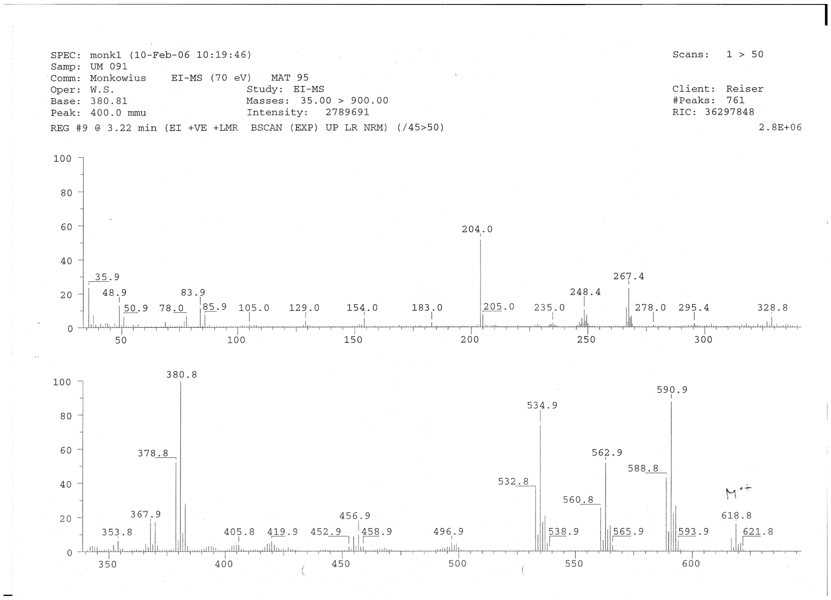

Figure S17: MS data for **Re**$\mathbf{P\cap N}$

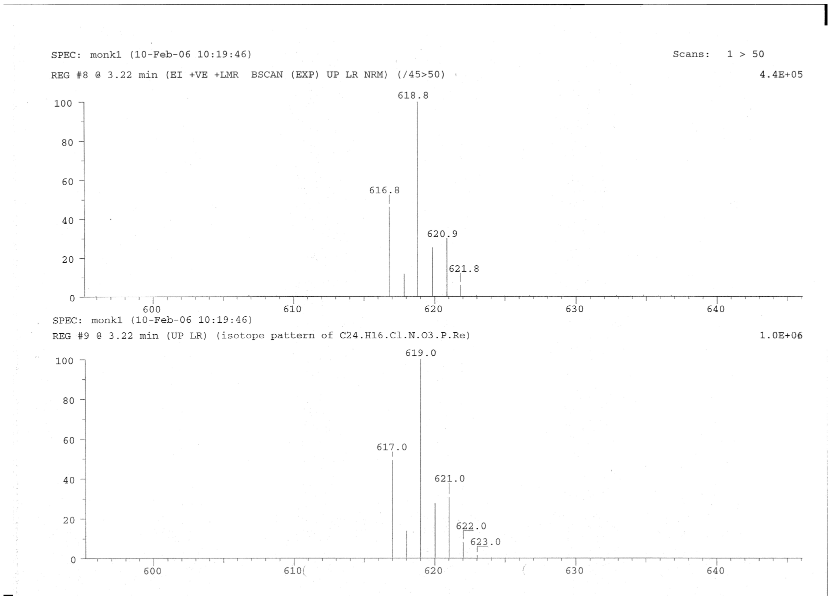

Figure S18: Calculated MS data for **Re**$\mathbf{P\cap N}$

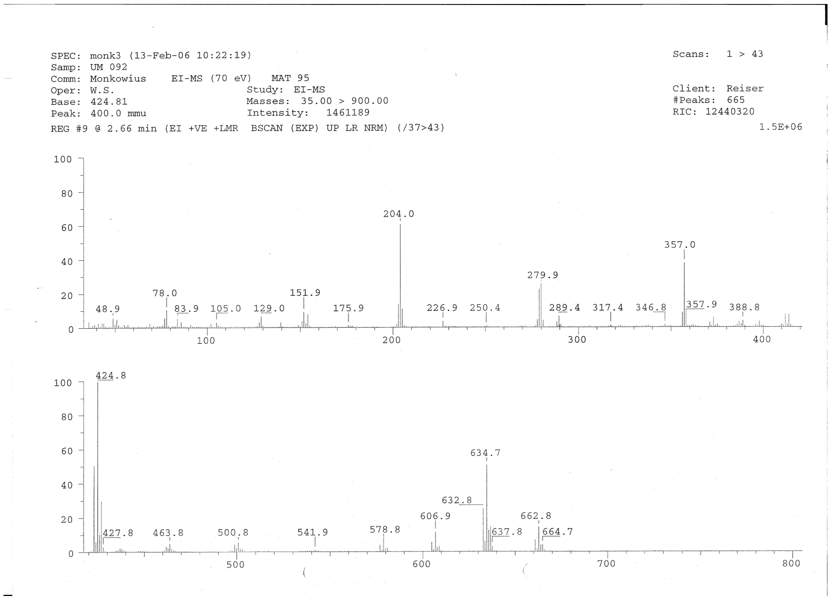

Figure S19: MS data for **ReAs∩N**

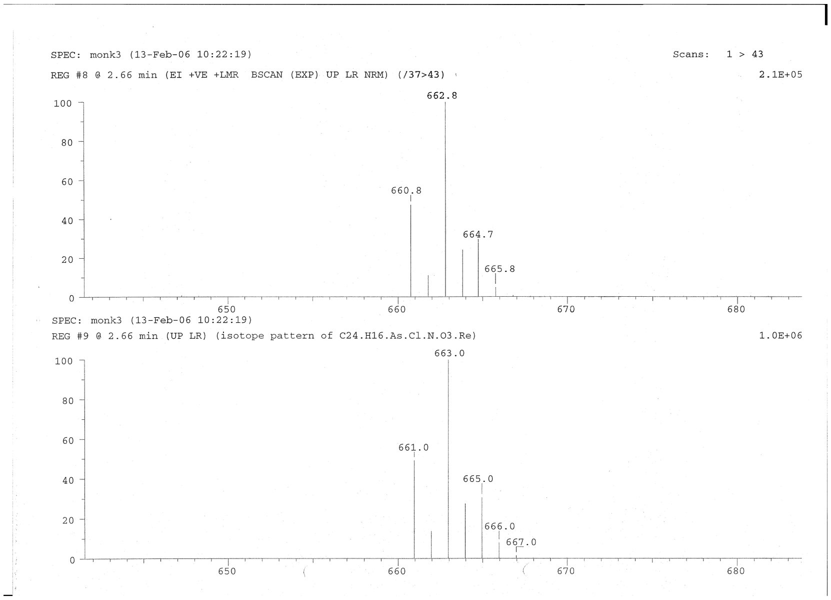

Figure S20: Calculated MS data for **ReAs∩N**

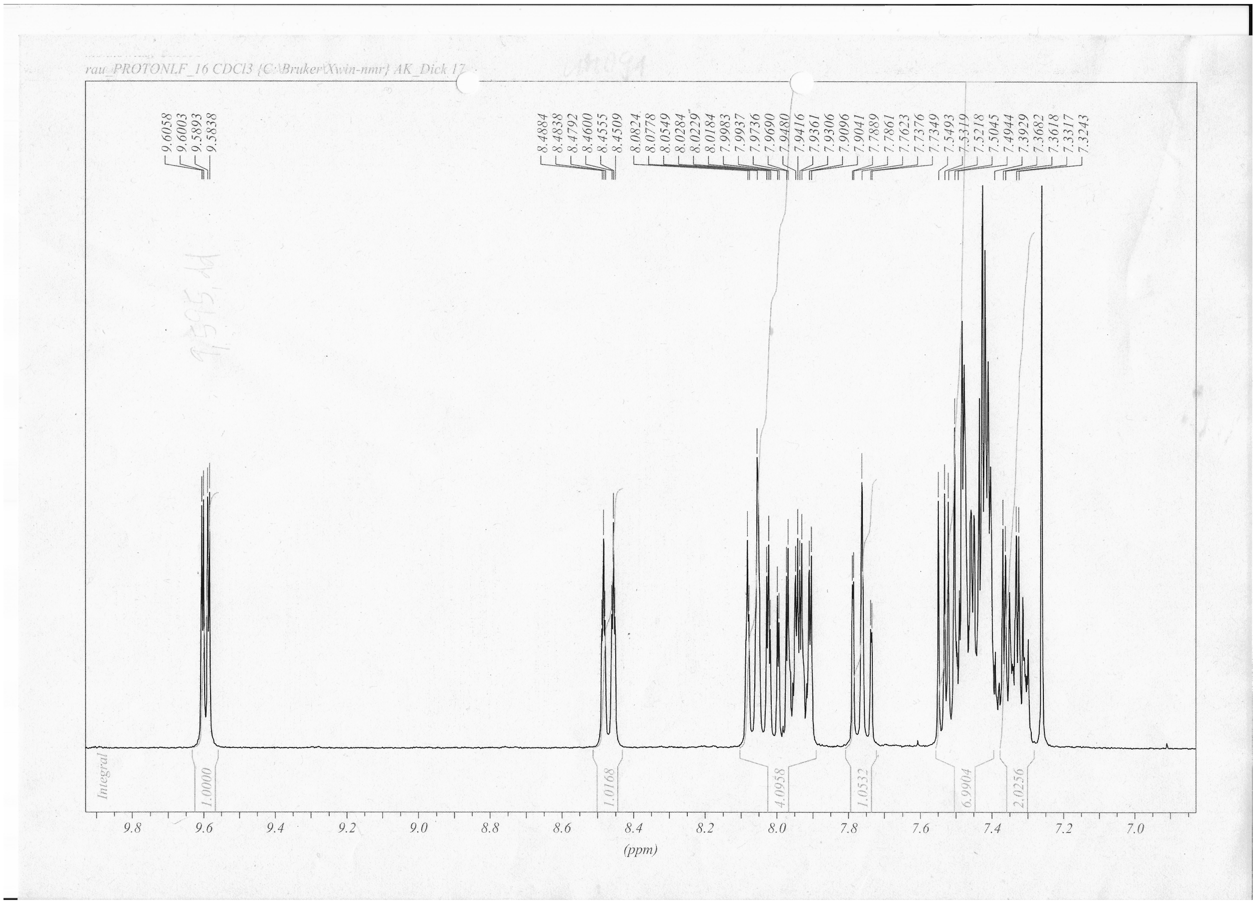

Figure S21: ^{1}H-NMR data for **ReP∩N**

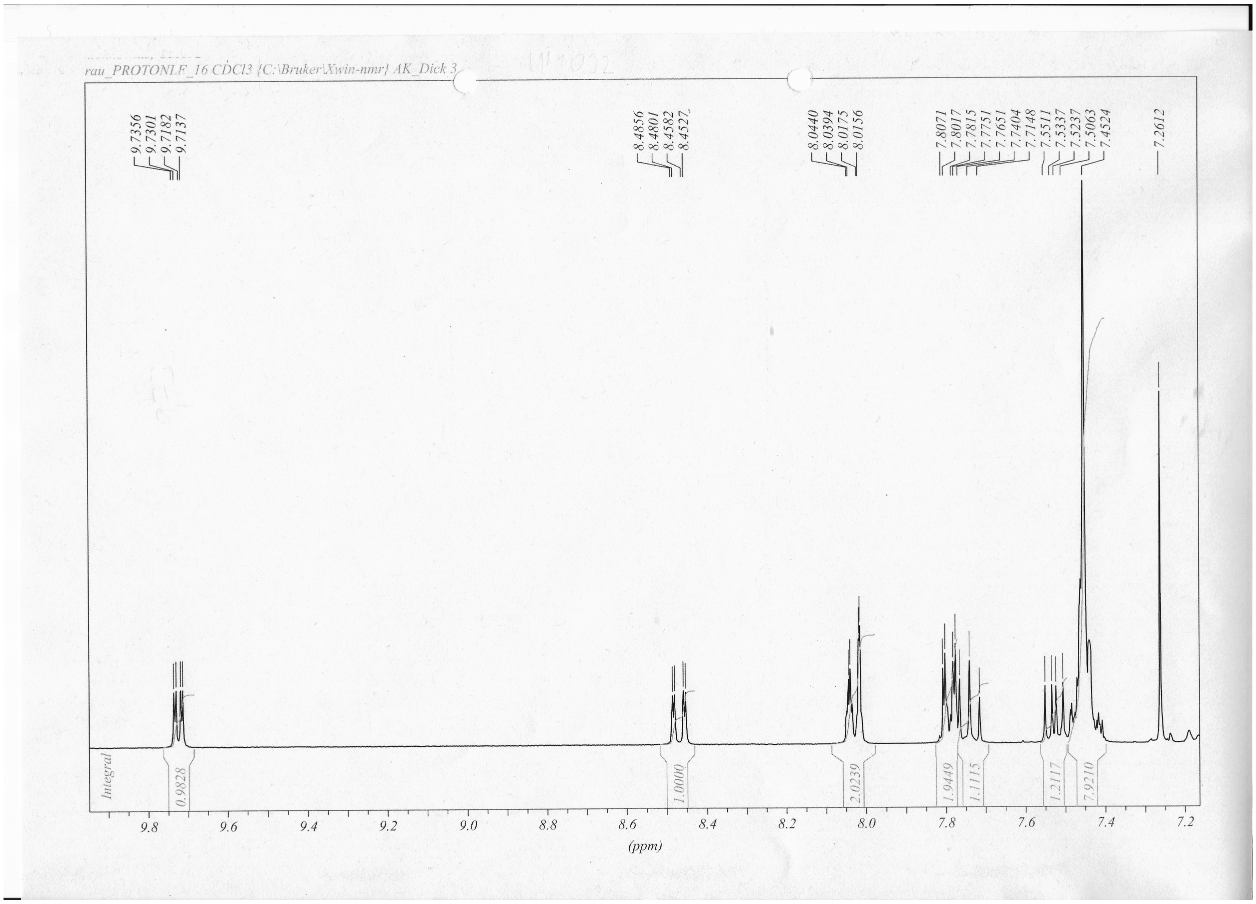

Figure S22: ^{1}H-NMR data for **ReAs∩N**

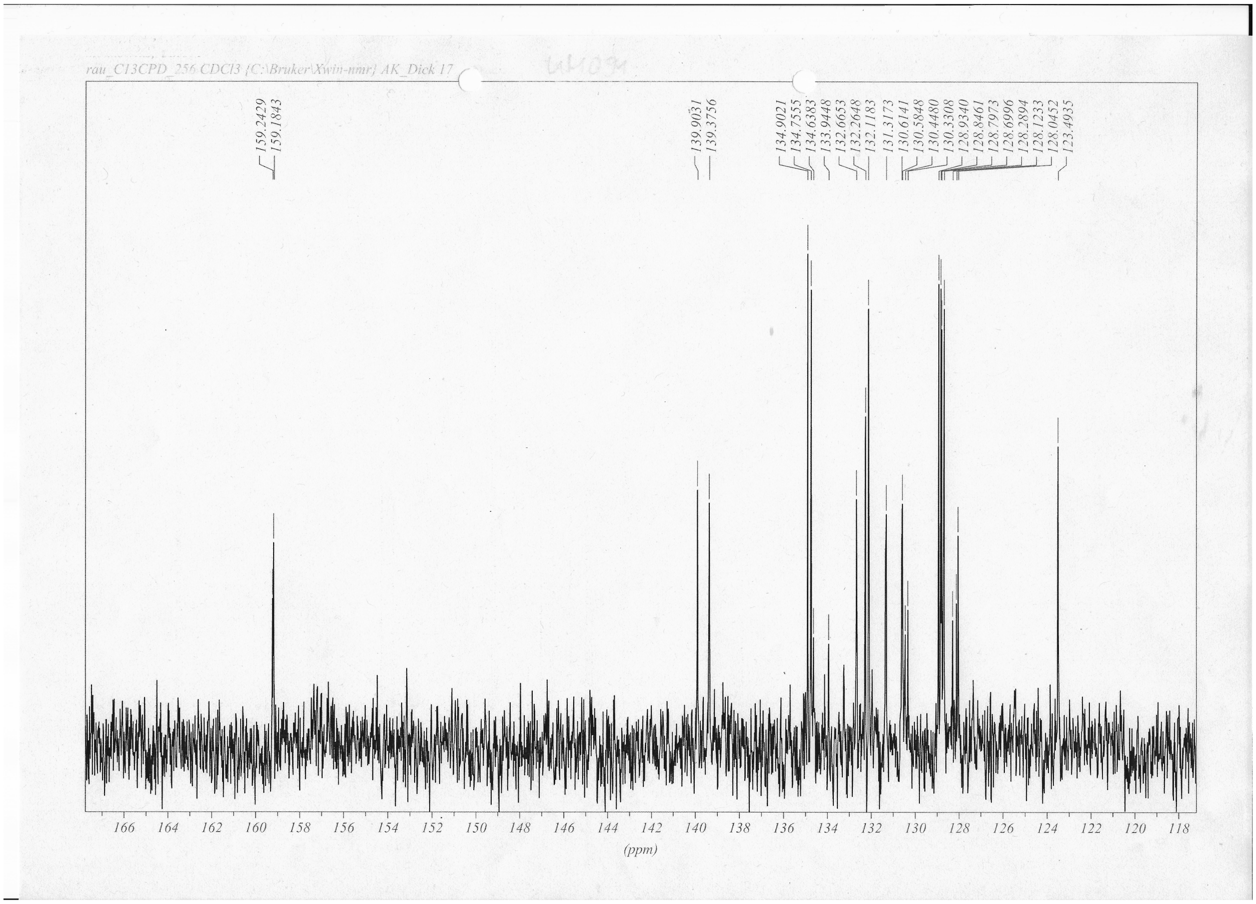

Figure S23: ^{13}C-NMR data for **ReP∩N**

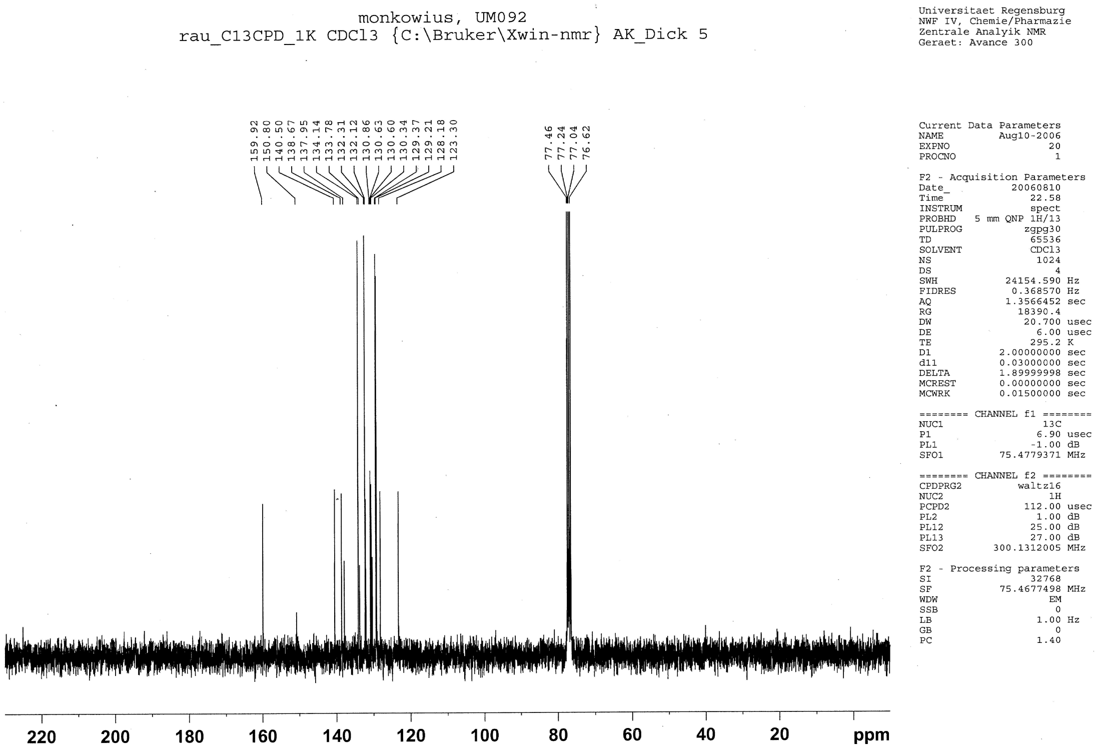

Figure S24: ^{13}C-NMR data for **ReAs∩N**

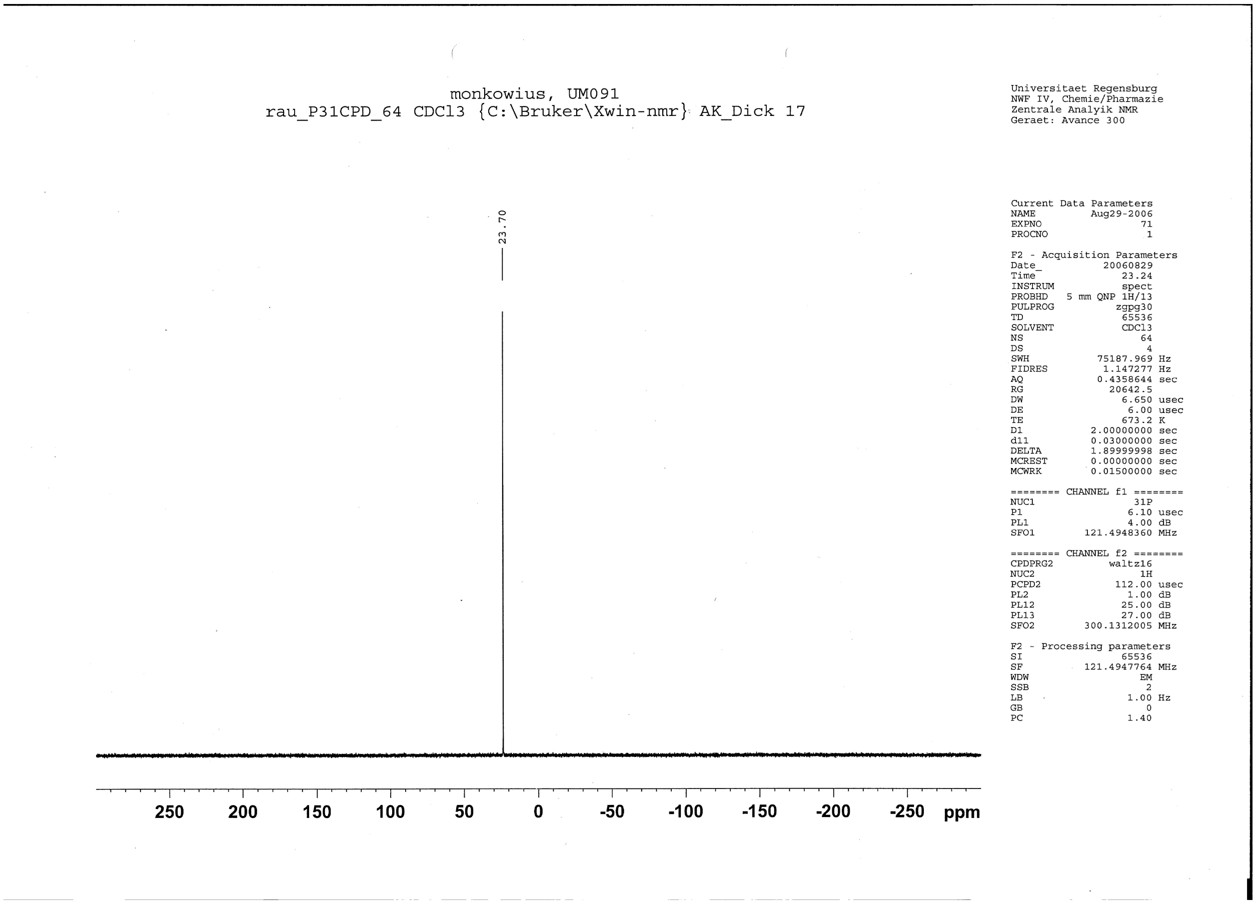

Figure S25: ^{31}P-NMR data for **Re$\mathbf{P\cap N}$**

References

(1) Krejčik, M.; Daněk, M.; Hartl, F. Simple construction of an infrared optically transparent thin-layer electrochemical cell. *Journal of Electroanalytical Chemistry and Interfacial Electrochemistry* **1991**, *317*, 179–187, DOI: 10.1016/0022-0728(91)85012-E.

(2) Pschenitza, M.; Meister, S.; Rieger, B. Positive effect of 1,8-diazabicyclo[5.4.0]undec-7-ene (DBU) on homogeneous photocatalytic reduction of CO_2. *Chemical Communications* **2018**, *54*, 3323–3326, DOI: 10.1039/C7CC08927A.
